# Smartphone-Tracked Digital Markers of Momentary Subjective Stress in College Students: Idiographic Machine Learning Analysis

**DOI:** 10.2196/37469

**Published:** 2023-03-23

**Authors:** George Aalbers, Andrew T Hendrickson, Mariek MP Vanden Abeele, Loes Keijsers

**Affiliations:** 1 Department of Cognitive Science & Artificial Intelligence Tilburg University Tilburg Netherlands; 2 Department of Communication and Cognition Tilburg University Tilburg Netherlands; 3 Media, Innovation and Communication Technologies Department of Communication Sciences Ghent University Ghent Belgium; 4 Clinical Child and Family Studies Erasmus School of Social and Behavioural Sciences Erasmus University Rotterdam Rotterdam Netherlands

**Keywords:** mobile health, mobile phone, digital phenotype, digital biomarker, machine learning, personalized models

## Abstract

**Background:**

Stress is an important predictor of mental health problems such as burnout and depression. Acute stress is considered adaptive, whereas chronic stress is viewed as detrimental to well-being. To aid in the early detection of chronic stress, machine learning models are increasingly trained to learn the quantitative relation from digital footprints to self-reported stress. Prior studies have investigated general principles in population-wide studies, but the extent to which the findings apply to individuals is understudied.

**Objective:**

We aimed to explore to what extent machine learning models can leverage features of smartphone app use log data to recognize momentary subjective stress in individuals, which of these features are most important for predicting stress and represent potential digital markers of stress, the nature of the relations between these digital markers and stress, and the degree to which these relations differ across people.

**Methods:**

Student participants (N=224) self-reported momentary subjective stress 5 times per day up to 60 days in total (44,381 observations); in parallel, dedicated smartphone software continuously logged their smartphone app use. We extracted features from the log data (eg, time spent on app categories such as messenger apps and proxies for sleep duration and onset) and trained machine learning models to predict momentary subjective stress from these features using 2 approaches: modeling general relations at the group level (*nomothetic approach*) and modeling relations for each person separately (*idiographic approach*). To identify potential digital markers of momentary subjective stress, we applied explainable artificial intelligence methodology (ie, Shapley additive explanations). We evaluated model accuracy on a person-to-person basis in out-of-sample observations.

**Results:**

We identified prolonged use of messenger and social network site apps and proxies for sleep duration and onset as the most important features across modeling approaches (nomothetic vs idiographic). The relations of these digital markers with momentary subjective stress differed from person to person, as did model accuracy. Sleep proxies, messenger, and social network use were heterogeneously related to stress (ie, negative in some and positive or zero in others). Model predictions correlated positively and statistically significantly with self-reported stress in most individuals (*median person-specific correlation*=0.15-0.19 for nomothetic models and *median person-specific correlation*=0.00-0.09 for idiographic models).

**Conclusions:**

Our findings indicate that smartphone log data can be used for identifying digital markers of stress and also show that the relation between specific digital markers and stress differs from person to person. These findings warrant follow-up studies in other populations (eg, professionals and clinical populations) and pave the way for similar research using physiological measures of stress.

## Introduction

### Background

Stress is an important predictor of mental health problems such as burnout [1] and depression [2]. How stress influences mental health depends on its duration. Stress with a duration of minutes to hours (*acute stress*) is commonly considered an adaptive psychophysiological response, whereas stress lasting for weeks to months or even years (*chronic stress*) is believed to have adverse psychological and physiological consequences [3]. Given its potential effects, early detection and treatment of chronic stress is important to prevent mental health problems.

As asking individuals to consistently self-monitor and self-report stress over extended periods is a difficult, costly, and time-intensive procedure [4], researchers have started developing algorithms to unobtrusively detect stress from passively logged data, such as smartphone app use log data [5]. If successful, such algorithms open opportunities for early detection of the tipping point where acute stress turns into chronic stress, possibly unlocking earlier possibilities for scalable interventions, such as smartphone-based cognitive behavioral therapy with chatbots [6].

Previous research suggests that smartphone use log data (among other passively logged data sources) might be used to recognize how stressed a person feels [5,7-10], with pioneering studies in college students using call and SMS text messaging log data (among others) as predictors [9,10]. However, following technological advances that made smartphones more powerful, use of these devices has evolved, and college students now typically access their smartphone for activities other than calling or SMS text messaging, such as using social media [11]. As a result, more research is required to identify whether stress might be recognized using smartphone app use patterns with high relevance to the current generation of students. We, therefore, extend previous work in the domain [5,7-10] to a large sample of contemporary college students and explore to what extent machine learning models can leverage features of smartphone app use to recognize momentary subjective stress and which of these app use features are most important for predicting momentary subjective stress and represent potential *digital markers* of stress. Inspired by recent findings in clinical psychology [11,12] and communication science [13-16], we then shed light on the nature of the relations between these potential digital markers and stress and the degree to which these relations differ from one person to another. Following related machine learning research on mood recognition [17], we also assess how important digital markers are relative to temporal features: time of day, day of the week, day of the month, and before COVID-19 versus during COVID-19 lockdown.

### Digital Markers

Digital markers are digital footprints, such as features of smartphone use log data, that are related to psychological or biological states [18], such as stress. Such features might represent any quantification of raw log data of digital devices, ranging from simple (eg, time spent using a device) [19] to more complex (eg, daily life patterns derived from device use) [20]. In this study, we investigate two types of potential digital markers of stress: (1) use of different types of smartphone apps (eg, duration and frequency of social network, messenger, and video apps) and (2) sleep proxies derived from smartphone app log data.

### Smartphone Use Behaviors as Potential Digital Markers

Smartphones enable individuals to perform a wide range of behaviors with profound psychological meaning and relevance to momentary subjective stress. Extant evidence, however, suggests a complex and nuanced relation between smartphone use and mental health, with different types of app use showing different patterns of association. For example, smartphone use behaviors with particular theoretical relevance to stress are calling, mobile messaging, and using social media, but recent work on passively logged data found depression relates negatively to calling [21], whereas it relates positively to social media use [22]. Moreover, research suggests that relations between smartphone app use and mental health differ from person to person [12-16]. Altogether, with respect to the association between smartphone use and mental health, these and other findings [23,24] indicate that smartphone behaviors could be informative about stress, but open questions remain.

### Sleep Proxies as Potential Digital Markers

Smartphone log data not only captures smartphone behavior with relevance to stress but might also be used to quantify sleep, which is a universal behavior related to stress [25]. As the human sleep-wake cycle closely aligns with smartphone app use patterns, different disciplines have leveraged smartphone log data (eg, call records, screen on-off status, and screen taps) to estimate sleep onset, offset, and duration [20]. To explore if such sleep proxies might be useful for stress recognition, we applied a rule-based algorithm (similar but not identical to a recent study by Massar et al [20]) to extract proxies for sleep duration and sleep onset from smartphone app log data and included these as features in our models.

### Explainable Artificial Intelligence

One potential avenue to identify digital markers is to (1) train machine learning models to find a mathematical mapping from digital markers to self-reports of momentary subjective stress and (2) apply explainable artificial intelligence (eg, Shapley values [26]) to clarify how these models make predictions. Applying explainable artificial intelligence is necessary because the structure of some powerful models (eg, random forest [RF]) preclude the straightforward interpretation of parameters that linear statistical models have.

By taking this approach, we aim (1) to identify digital markers by testing which features of smartphone use log data a model uses to predict momentary subjective stress and (2) to understand the nature of the relation between these digital markers and stress. For instance, time spent on messenger apps might be important for the prediction of stress and negatively related to stress, suggesting that individuals tend to spend less time on these apps when feeling stressed. This could potentially indicate that stress reduces social interaction or vice versa.

### Nomothetic Versus Idiographic

When training machine learning models on human-subjects data, it is important to take into account that individuals differ from another. Hence, both machine learning [27] and behavioral scientists [28] have underlined the importance of ensuring that models of human-subjects data are applicable to the individuals they pertain to. This is certainly also important in the domain of digital markers of stress. Behavioral scientists have shown that relations between digital trace data and psychological self-reports differ across individuals [19]. In parallel, machine learning researchers have demonstrated that personalized models, which are known as idiographic models in behavioral science, tend to predict subjective stress more accurately than nonpersonalized models [27], which are also referred to as nomothetic models. Naturally, these findings go hand in hand: a personalized (idiographic) model will more adequately capture person-specific dependencies between digital trace data and stress and therefore should make more accurate predictions than a nonpersonalized (nomothetic) model. In this study, we implement, evaluate, and compare both approaches.

### Objectives

This study has four complementary aims to explore: (1) to what extent machine learning models can leverage features of smartphone app use log data to recognize momentary subjective stress in individuals, (2) which of these features are most important for predicting stress and represent potential digital markers of stress, (3) the nature of the relations between these digital markers and stress, and (4) the degree to which these relations differ from one person to another.

## Methods

### Participants

We followed reporting guidelines recommended for experience sampling studies [29]. For a preregistered data collection [30], we used the university participant pool to recruit 247 student participants with an Android operating system on their primary phone, 224 (90.7%) of whom we included for analysis. Their average age was 21.97 (SD 3.04) years and the majority were female (125/224, 55.8%). We excluded (1) participants with operating systems other than Android on their primary phone and (2) participants with insufficient survey responses for training idiographic machine learning models (<6). Most participants (186/224, 83%) started participation before the first (reported) local infection of SARS-CoV-2 and a minority (38/224, 17%) after. With the exception of 2 participants, all participants had been active in our study before the first nation-wide lockdown. Throughout the study, the original (Wuhan) strain of SARS-CoV-2 was dominant.

### Procedure

#### Ethics Approval

Ethics approval was issued by the Tilburg University Ethics Committee (approval code REDC 2019/94c).

#### Onboarding

Participants were recruited through the university participant pool. After receiving web-based information through Qualtrics (Qualtrics XM), having been offered a possibility to ask questions, and signing an informed consent form, participants followed web-based instructions to install 2 apps on their smartphone. After completing these instructions, the participants attended an onboarding session in which we provided additional information, offered further opportunity to ask questions, and motivated the participants to participate to the best of their ability.

#### Technology

All participants installed 2 apps on their Android device: Ethica Data [31] and mobileDNA [32]. Ethica Data is an app that prompts participants to complete brief surveys on their smartphone (ie, *experience sampling*). MobileDNA is an app that unobtrusively logs a person’s smartphone app use, smartphone notifications, and location (ie, *passive logging*).

#### Sampling Scheme

The data collected in this study are part of a larger research project with other questions that required a so-called measurement burst design (1 period of intensive data collection followed by a break followed by another period of intensive data collection) [33]. The important advantage of this procedure is that it reduces participant burden, while capturing information on a larger timescale. Hence, in a 4-month period, Ethica notified the participants 5 times a day for a maximum of 60 days (30 days in month 1 and 30 days in month 4) at pseudorandom times between 8:30 AM and 10:30 PM to complete a 10-item survey (approximately 1 minute to complete) on stress and other constructs (fatigue, procrastination, and mood), whereas mobileDNA continuously logged smartphone app use. Following an initial push notification, each survey was available to the participant for 50 minutes. After 45 minutes, they received a reminder notification. After 50 minutes, the survey expired. The participants were allowed to catch up on 1 missed survey per day by starting and completing a new survey.

#### Monitoring Protocol

We actively monitored participant compliance and motivated participants with weekly emails containing personalized feedback. When a participant failed to complete many consecutive surveys, we sent an email to inquire why they could not comply with the study protocol and how any issues might be resolved. In a limited number of cases, the participants did not respond to such emails, in which case we contacted them through a phone call. The participants were compensated with course credits for research participation and were entered into a raffle comprising 20 prizes of 15 euros (US $22.06).

#### Compliance

**Figure 1:**
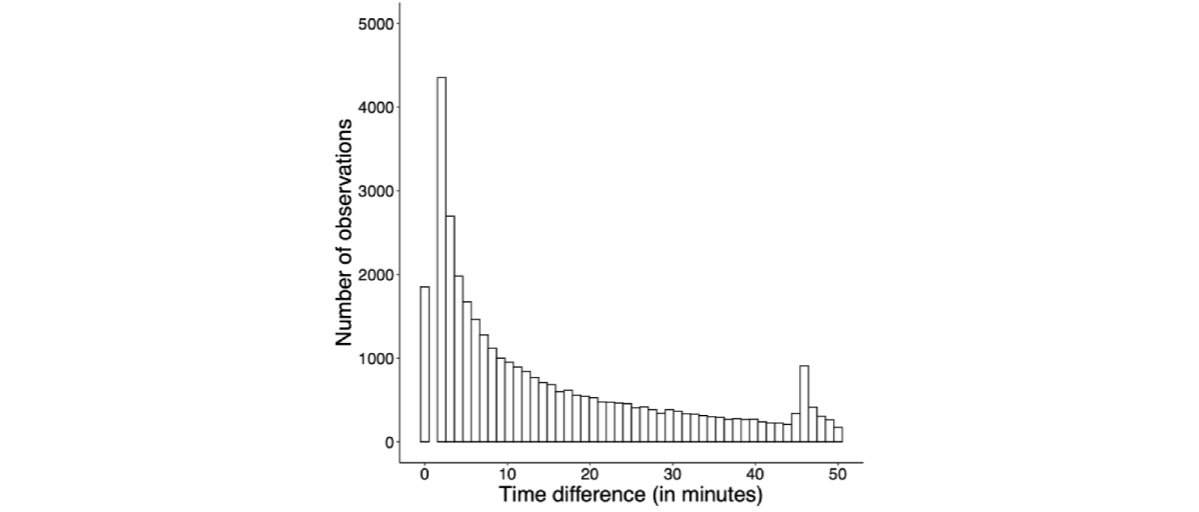
Distribution of the time difference between receiving the initial notification and completing the survey (in minutes).

**Figure 2:**
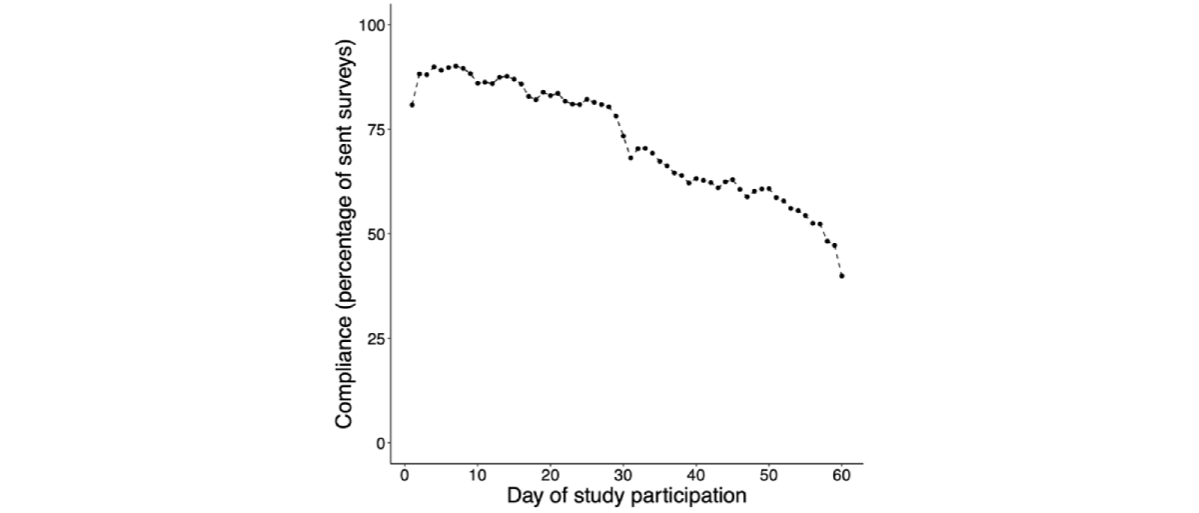
Daily compliance rate over time. To determine daily compliance rate, we computed the percentage of survey responses that were received on a given participation day relative to the total number of surveys to be sent each day based on our original sample size (224×5=1120). If, for instance, all participants completed 4 out of 5 surveys on their first day of participation, daily compliance equals 80%.

The participants (N=224) completed a total of 44,381 surveys (198 per person on average). Though data collection spanned the introduction of the COVID-19 pandemic lockdown, the median participant remained in the study across 4 months. In the sample we analyzed, the median participant completed 205 surveys (SD 87.42) and had 3072 hours of smartphone use log data. The median time difference between receiving the initial notification and completing the survey was 6 minutes (SD 14.26; refer to Figure 1 for a histogram). Reasons for noncompliance ranged from technical difficulties (eg, not receiving any notifications and broken or lost smartphone) to not being able to complete a survey (eg, waking up too late and receiving a notification during work or lecture) to personal reasons (eg, attrition because of COVID-19 pandemic–related personal problems or collecting sufficient course credits). Figure 2 visualizes how compliance rate changed from the first to the last day of the study.

### Measures

To measure participants’ current (ie, in-the-moment) subjective level of stress, we used the Stress Experience Sampling Scale [34]. This scale consists of 2 items on a 7-point Likert scale ranging from 1 (not at all) to 4 (moderately) to 7 (very much): “Right now, I feel relaxed” and “Right now, I feel stressed (tense, restless, nervous or anxious).” The 2 items have an adequate intraclass coefficient (>50% of variance because of within-person fluctuations) and acceptable within-person reliability as assessed by within-person omega (ω=.71). We calculated an unweighted average of the 2 items and subtracted each participant’s average level of stress from the resulting values (ie, within-person centering).

Textbox 1 provides an overview of the features included in this study. We analyzed three categories of features: (1) smartphone use behavior, (2) sleep, and (3) time. As the raw values of these features are on vastly different scales, which can dramatically impact model performance, we scaled all features to a range between 0 and 1 using MinMaxScaler in *sklearn* [35], based on the minimum and maximum values in the training data.

To extract time spent on different smartphone app categories, we first categorized apps using a coding scheme that maps app names (eg Whatsapp [36]) to 1 of the 18 major categories (Textbox 2; these core categories represent 4,046,581/5,277,494, 76.68% of all app events). Then, during the 60 minutes before each self-report of stress, we calculated (1) the total time spent on all apps in a category (*duration*) and (2) the total number of times apps in a category had been accessed (*frequency*).

To extract sleep duration and sleep onset proxies from the raw smartphone app log timestamps, we used an algorithm similar (but not identical) to a previously validated rule-based algorithm [11] (refer to Multimedia Appendix 1 for a description of our approach), an algorithm that was found to be strongly associated with actigraphy-based and self-reported sleep duration and onset.

To extract time features (ie, hour of the day, day of the week, day of the month, and lockdown status) from the raw self-report timestamps, we used pandas.datetime in Python 3.9.9 [37]. In the interest of model simplicity, we recoded day of the week into a binary variable (weekday=0 and weekend=1) rather than treating this variable as a categorical variable. We further coded lockdown status as a binary variable (before COVID-19 lockdown=0 and during COVID-19 lockdown=1) based on the lockdown timing in the Netherlands.

**Overview of the features included in models.**

**Smartphone use behavior**

- Time (seconds) spent on smartphone app category X in the past 60 minutes
- Frequency (count) of opening smartphone app category X in the past 60 minutes

**Sleep**

- Sleep onset (hours and postmidnight hours >24)
- Sleep duration (hours)

**Time**

- Hour of the day (0 to 23 and starting at midnight)
- Day of the week (0=weekday and 1=weekend)
- Day of the month (0 to 31)
- COVID-19 (before lockdown=0 and during lockdown=1)

**Smartphone app categories and examples of apps per category.**

- Browser: Chrome and Opera
- Calling: default dial apps
- Camera: default camera apps
- Dating: Tinder and Grindr
- Email: Gmail and Outlook
- Exercise: RunKeeper
- Food and drink: UberEATS
- Gallery: default gallery apps
- Game: CandyCrush
- Messenger: WhatsApp
- Music and audio: Spotify
- Productivity: Microsoft Word
- Shared transportation: 9292OV (Dutch public transport)
- Social network: Facebook, Instagram, and Twitter
- Tracker: pedometer apps
- Video: YouTube and Netflix
- Weather: default weather apps
- Work: StudentJob and EmployeeApp

### Stress Recognition Models

#### Model Types

We trained 3 different types of machine learning models: least absolute shrinkage and selection operator (LASSO) regression, support vector regression (SVR), and RF regression. We trained all models using a nomothetic and an idiographic approach (explained in the section Model Cross-Validation). For brevity and clarity, we prepend an “N” to the abbreviations of our nomothetic models (ie, N-LASSO, N-SVR, and N-RF) and an “I” to the abbreviations of our idiographic models (ie, I-LASSO, I-SVR, and I-RF).

#### Model Cross-Validation

Generally, when we perform cross-validation (CV), we (1) split the data into train and test data, (2) specify a range of values for a model’s different hyperparameters (ie, researcher-specified parameters that control model complexity), (3) identify the best hyperparameters per model using *k*-fold CV within the training set, and (4) evaluate each model based on predictive accuracy for the test data. In what follows, we outline how we cross-validated nomothetic and idiographic models.

To train nomothetic models, we applied a user split to distinguish between train and test data. We first used group *k*-fold CV (GroupKFold in *sklearn* [35]) to partition the data into 5 subsets. A total of 4 subsets contained data from 45 participants, and 1 subset contained data from 44 participants. We then selected 4 subsets (*train data*) to train a model. For training the model, we used 5-fold grid search CV (GridSearchCV in *sklearn* [35]) to minimize each model’s default *sklearn* error metric (refer to Multimedia Appendix 2 for tuned hyperparameters and minimized error metrics). After training the model, we let the model make predictions on the data subset we did not include in training (*test data*; ie, all observations of participants excluded from training). Finally, we evaluated the accuracy of these predictions. We repeated this process until each subset of the data had been left out of training once and did so for all models.

To train idiographic models, we applied a time split to distinguish between train and test data. We iteratively selected 1 participant’s data to train and test models only on these data. For each person, we assigned each participant’s first 80% observations to a train data set and their final 20% observations to a test data set and trained each model (SVR, RF, and LASSO). We applied 5-fold grid search CV to each participant’s train data to optimize hyperparameters. As the number of idiographic models to train is much larger, we applied 5-fold randomized search CV rather than grid search CV for training RFs, which are more computationally expensive than LASSO and SVR. Grid search CV always uses a larger number of hyperparameters than randomized search CV because the latter trains models on a random subset of all the hyperparameter settings used by the former. Randomized search CV considerably speeds up training time and makes training a large number of RF models more feasible. Finally, we let trained models make predictions on the individual’s test data (ie, final 20% observations) and evaluated the accuracy of these predictions.

#### Model Evaluation

To evaluate the accuracy of models, we used Spearman rho rank-order correlation and mean absolute error (MAE) as evaluation metrics. Spearman rho rank-order correlation indicates whether a model tends to predict greater values when an individual feels more stressed without assuming a linear relation. We consider a model to perform above chance for a given individual if the Spearman ρ between predictions and self-reports between predictions and self-reports has a *P* value below .05.

Lower MAE values indicate a more accurate model. We consider the MAE to be an intuitive metric to assess predictive accuracy on a target variable measured on a 7-point Likert scale, as it allows us to make statements such as “on average, the model mispredicts momentary subjective stress by ±0.80 points on a 7-point Likert scale).” To evaluate if our models perform better than random guessing, we compare against the MAEs of a “naive” but person-specific baseline model. This “naive” baseline model always predicts an individual’s average level of stress.

#### Model Explanation

One of the challenges of machine learning approaches is to understand the results, as they are more complex and therefore less intuitive than standard statistical approaches. To explain models, we use the Shapley additive explanations (SHAP) library [26] implemented in Python 3.9.9 [37] to calculate and visualize Shapley values. Shapley values can be used to determine (1) which features are most important in a model and (2) how features are related to model predictions.

## Results

### Recognizing Momentary Subjective Stress

Table 1 provides an overview of how accurately nomothetic models predict out-of-sample data. Model predictions correlated positively and significantly with self-reports in a majority of participants for N-LASSO (116/224, 51.3%), N-SVR (120/224, 53.6%) and N-RF, with N-RF performing best in terms of percent significant results (124/224, 55.4%). The median correlation between predictions and self-reports was weak for all models (between 0.15 and 0.19). Correlations also differed between participants: in 60 participants, the positive correlation was moderate or larger (ρ>0.3), whereas it was negative (range *P*<.001 to *P*=.049) in 2.2% (5/224) of people. In the median participant, models on average mispredicted momentary subjective stress by approximately 0.8 points on a 7-point Likert scale (MAE of 0.84; scale range: 1=“Not at all,” 4=“Moderately,” and 7=“Very much”). The MAE of nomothetic models varied across individuals, but for 89.3% (200/224) of participants, the person-specific baseline outperformed all nomothetic models (refer to the sixth column in Table 1), followed by N-LASSO (20/224, 8.9%) and N-SVR (4/224, 1.8%). N-RF did not outperform the other models in terms of MAE.

Thus, nomothetic models make predictions that weakly and positively correlate with actual stress self-reports for the majority of participants (up to 124/224, 55.3%). This means that when these participants, who were not included in the training data set, feel stressed, the model tends to output a higher value, and when a participant does not feel stressed, the model tends to output a lower value. However, these models are not highly accurate, as they often do not outperform a person-specific baseline. For most participants, the association between predictions and actual self-reported stress is positive, but it is significantly negative (range *P*<.001 to *P*=.049) for a very small group (5/224, 2.2%). Thus, for a very small group of participants, the model tends to detect subjective stress when the person does not feel stressed, and vice versa.

We then evaluated how well idiographic models recognize momentary subjective stress in out-of-sample observations. For all models, model predictions of momentary subjective stress correlated positively and significantly with self-reports of momentary subjective stress, but only in a minority of participants. In 60 participants, this correlation was moderate or larger (ρ>0.3), whereas the overall median correlation was absent to weak (range of median correlations per model, ρ*=*0.00-0.09). In a minority (11/224, 4.9%), model predictions of stress and self-reported stress were significantly negatively associated. Similar to the nomothetic models, the median person-specific MAE for each idiographic model was slightly >0.8 points. These MAE scores are compared with a person-specific baseline based on the person-specific average level of stress in the participant’s train data (ie, first 80%) rather than all their data to prevent data leakage from train to test data. MAE varied from individual to individual, but for 80.4% (180/224) of participants at least one of the idiographic models outperformed the person-specific baseline. The I-SVR most frequently outperformed all other models (including person-specific baseline; 92/224, 41.1%), followed by I-RF (47/224, 20.9%) and I-LASSO (43/224, 19.2%).

**Table 1:** Central tendency and range of out-of-sample predictive accuracy (Spearman ρ correlation and mean absolute error [MAE]) for nomothetic and idiographic models on a person-by-person basis.

Model	Spearman ρ rank-order correlation		MAE		
	Median (range)	Percent significant	Median (range)	Better than baseline (%)	Best model (%)
N^a^-baseline	N/A^b^	N/A	*0.83*^c^ (0 to 3.39)	N/A	*89.29*
N-LASSO^d^	0.15 (−0.45 to 0.65)	51.34	0.84 (0.11 to 2.04)	10.27	8.93
N-SVR^e^	0.16 (−0.31 to 0.64)	53.57	0.84 (0.17 to 2.04)	4.91	1.79
N-RF^f^	*0.19* (−0.37 to 0.57)	*55.34*	0.84 (0.26 to 2.04)	3.57	0
I^g^-baseline	N/A	N/A	*0.83* (0 to 3.40)	N/A	19.64
I-LASSO	0.00 (−1 to 1)	13.84	0.87 (0 to 3.36)	34.37	18.75
I-SVR	*0.09* (−0.79 to 1)	*23.21*	0.84 (0 to 3.49)	*50.45*	*41.07*
I-RF	0.08 (−1 to 1)	19.64	0.84 (0 to 3.27)	39.73	20.98

^a^N: nomothetic.

^b^N/A: not applicable.

^c^Italicized values represent best performance.

^d^LASSO: least absolute shrinkage and selection operator.

^e^SVR: support vector regression.

^f^RF: random forest.

^g^I: idiographic.

### Identifying Digital Markers of Momentary Subjective Stress

**Figure 3:**
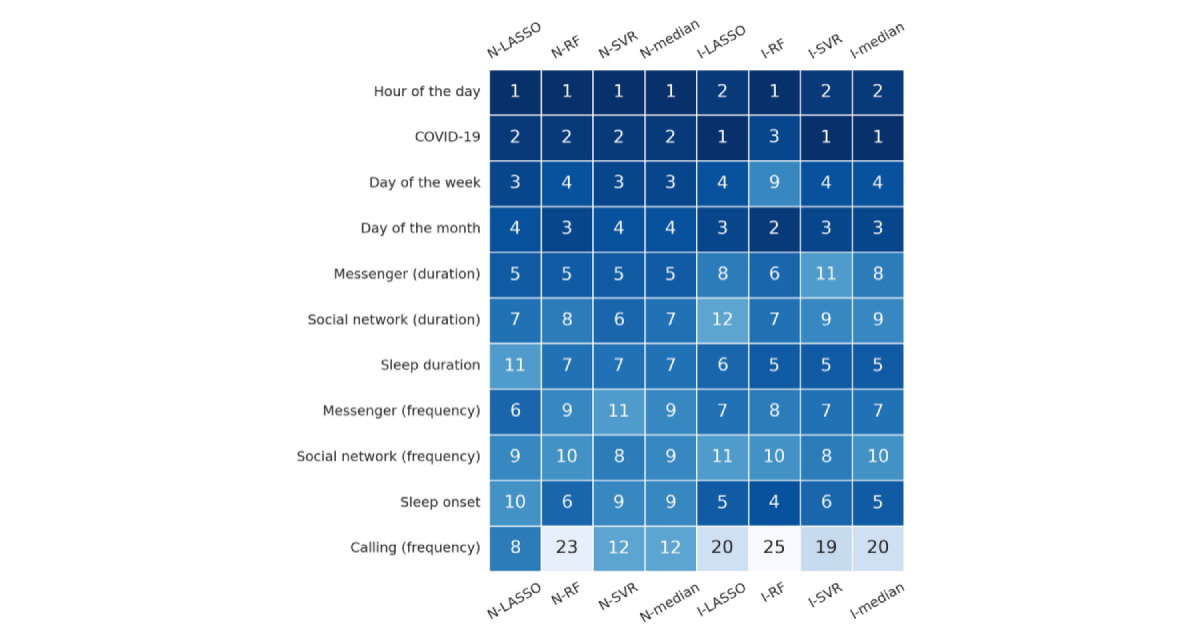
Feature importance ranking for each nomothetic (N) and idiographic (I) model, containing features that appeared in the top 10 features of any model. Numeric values represent the ranking of 1 feature for 1 model. Dark (top) cells represent more important features. N-median and I-median represent the median ranking of a feature across N and I models, respectively, where double values indicate a tie between 2 features. Features are ordered by N-median scores. LASSO: least absolute shrinkage and selection operator; RF: random forest; SVR: support vector regression.

To demonstrate which aspects of the model have the strongest predictive value, Figure 3 provides an overview of the 10 most important features per nomothetic and idiographic model. In all 6 models, temporal features, COVID-19 lockdown status, messenger and social network use, and sleep proxies were most important to the prediction stress. The largest disagreement between nomothetic and idiographic models was the importance of messenger and social network use relative to sleep duration and onset. The former features were more important in nomothetic models, whereas the latter were more important in idiographic models. Feature importance was relatively consistent for models across data splits (refer to Multimedia Appendix 3 for beeswarm plots per model per data split).

### Understanding Digital Markers of Momentary Subjective Stress

**Figure 4:**
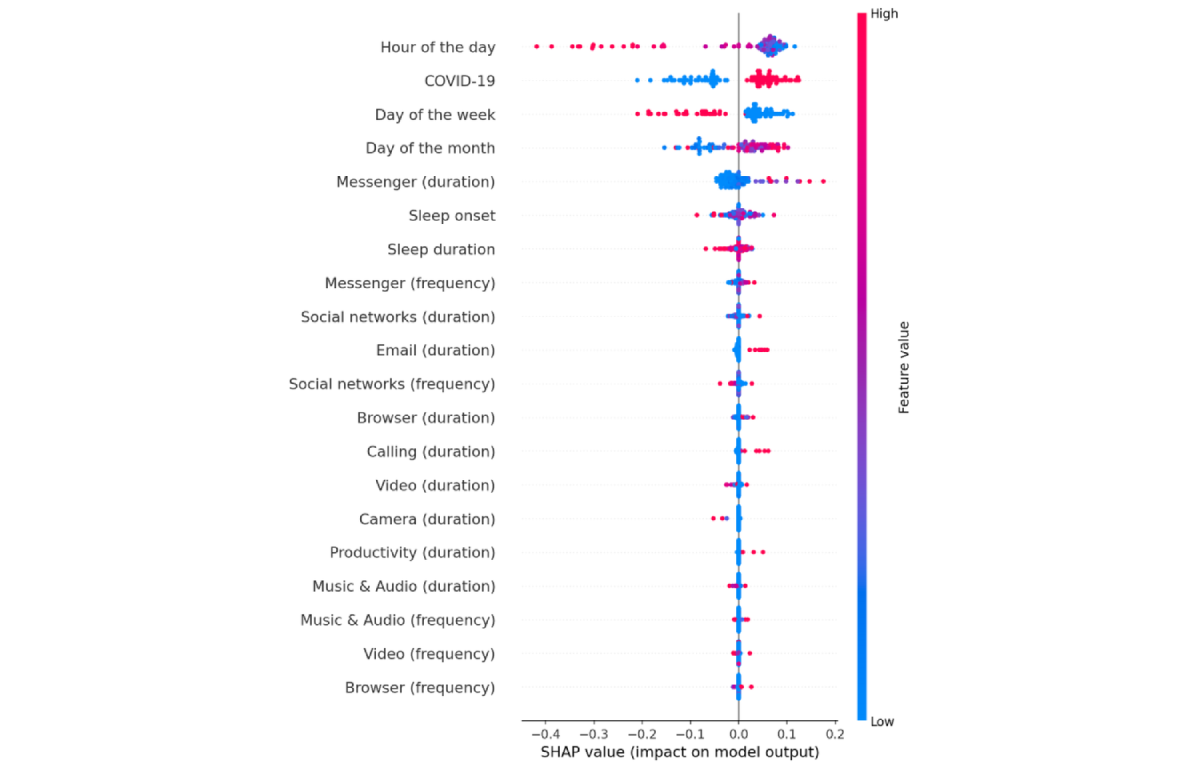
Shapley additive explanations (SHAP) beeswarm plot indicating the relative importance of each feature and the relation between feature values and model prediction for the nomothetic random forest model. COVID-19 is coded as 0=before lockdown and 1=during lockdown. Weekday is coded as 0=weekday and 1=weekend.

Feature importance provides relevant information about which features contribute most to predictions. However, it does not tell us whether small or large values of a feature are indicative of momentary subjective stress. For instance, although we have identified hour of the day as a relatively important predictor of momentary subjective stress, it is still unclear whether people tend to feel more stressed in earlier or later hours of the day. To clarify potential relations between features and momentary subjective stress, we present a beeswarm plot for the nomothetic RF (Figure 4; refer to Multimedia Appendix 3 for all beeswarm plots) to visualize how different features are related to model predictions in 1 split of the data. In this figure, for each feature (listed on the y-axis in decreasing order of importance), a point represents 1 test trial, the color of a point indicates the value of the feature (red for higher values [eg, later hour of the day] and blue for low values [eg, earlier hour of the day]), and the position along the x-axis indicates the SHAP value (positive values correspond with higher stress predictions; larger magnitude values indicate stronger impact). For any 1 feature, red points on the left side of the plot (high feature values and negative SHAP values) indicate a relation where increasing the feature value results in the model predicting lower outcome values (eg, higher hour predicts lower stress), whereas red points on the right side of the plot (high feature values and positive SHAP values) indicate a positive relation (eg, COVID-19 lockdown predicts higher stress). For instance, Figure 4 shows that N-RF predicts greater momentary subjective stress values (1) in earlier hours of the day (indicated in blue), (2) during COVID-19 lockdown, (3) on weekdays, and (4) later in the month. Similarly, when individuals spend more time on messenger (red), these models output greater values for momentary subjective stress.

### Interindividual Differences in the Relation Between Digital Markers and Stress

We also investigated whether the nature of relations between features and stress differed from person to person. As the zero-order Spearman rank-order correlations between features and stress are relatively weak (Multimedia Appendix 4), we instead calculated these correlations between feature values and SHAP values for each idiographic model. A positive correlation between feature and SHAP value indicates that when this feature has a higher value, the model predicts that an individual feels more stressed. SHAP values of more complex models have the potential benefit of capturing the nonlinear and interactive relations learned by the model. Correlations with *P* values of >.05 are not included.

**Figure 5:**
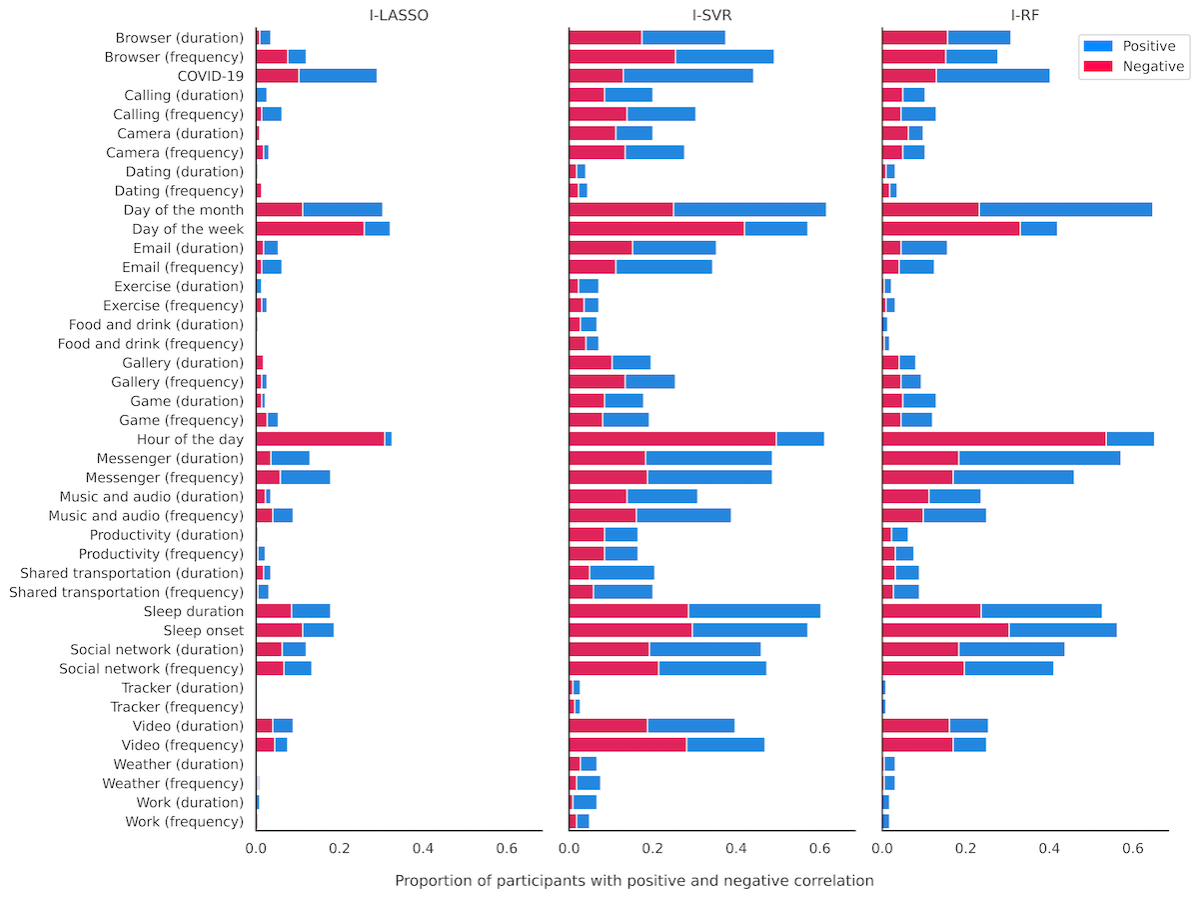
Stacked bar plots representing the proportion of participants showing a significant positive (blue bar) or negative (red bar) correlation between feature values and Shapley additive explanations (SHAP) values for each idiographic model. A positive correlation indicates that when a given feature has a higher value, then the model predicts that an individual feels more stressed. A negative correlation indicates that when a given feature has a higher value, then the model predicts that an individual feels less stressed. For instance, the idiographic random forest (I-RF) predicts a lower level of stress at later hours of the day in 53.4% (120/224) of individuals. I-LASSO: idiographic least absolute shrinkage and selection operator; I-SVR: idiographic support vector regression.

Figure 5 shows the frequency of significant positive and negative correlations between digital markers and SHAP values for the prediction of stress for each idiographic model. Interestingly, most bars are both red and blue, which indicates a relatively heterogeneous relation across people between that feature and stress for most features. For instance, there is much heterogeneity in the relation between stress and some of the most important features (Figure 3), including social network use and sleep duration. Some bars are mostly red or mostly blue, suggesting a relatively homogeneous relation across people between these features and stress. For instance, the relation between stress and hour of the day is mostly negative and the relation between stress and shared transportation app use is mostly positive. A few features rarely show any correlation with predicted stress, and this is likely because that model has learned to disregard those features or because the correlation between feature values and SHAP values is either not reliable or monotonic.

## Discussion

### Principal Findings

The aims of our study were to explore (1) to what extent machine learning models can leverage features of smartphone app use log data to recognize momentary subjective stress, (2) which smartphone app use features are most important for predicting stress and represent potential digital markers of stress, (3) the nature of the relations between smartphone app use features and stress, and (4) the degree to which these relations differ from one person to another. We found that when individuals were more stressed, the best performing nomothetic models tended to predict a higher level of stress (and vice versa) in the majority of individuals (up to 124/224, 55.3%). However, they generally did not predict stress with greater accuracy than a naive baseline model, which always predicted that a person was experiencing their average level of stress. We found these results to be similar for idiographic models, although these models predicted stress with greater accuracy than a naive baseline model. Although performance should be improved to make clinical application feasible, this study does suggest that, in the absence of self-report or physiological data, digital markers can be used to recognize momentary subjective stress on a person-by-person basis in out-of-sample data.

Using explainable artificial intelligence, we found that temporal features, prolonged messenger and social network app use, and smartphone-tracked sleep proxies were the most important features of the best-performing nomothetic and idiographic models. These models consistently ordered these features, with temporal features as most important and app use as less important drivers of stress predictions. The largest disagreement between nomothetic and idiographic models is the importance of app use duration. In nomothetic models, these are more important than sleep features, but in idiographic models, the order is opposite. In sum, though, prolonged use of messenger and social network apps and sleep proxies might be valid digital markers of stress.

Our results suggest that, for an average person, in the earlier hours of the day, on weekdays, on later days of the month, and during COVID-19 lockdown (compared with before COVID-19 lockdown), individuals felt more stressed than they would usually do. Individuals also felt more stressed when spending more time on social apps (ie, messenger and social network apps). The relation between temporal features and (SHAP values for the prediction of) stress was rather consistent across individuals, suggesting these could represent universal principles for this population. These may even be explained by biological mechanisms (eg, cortisol awakening response [38]) that universally affect adolescents and young adults. Such features are likely important to include in passive tracking in the context of stress-related mental health problems such as depression and burnout.

One of the unique features of this study was to compare nomothetic and idiographic models in light of increasingly idiographic research practices in behavioral science [29]. Our findings show that model personalization is warranted especially when smartphone app use features are added to the model. That is, the relation between social app use and stress was negative for some individuals and positive or absent for others (Figure 5). We, therefore, find evidence that the idiographic approach of digital phenotyping research, that is, “the moment-by-moment quantification of the individual-level human phenotype in situ using data from personal digital devices, in particular smartphones” [39], is warranted in the context of stress.

In clinical practice, the added value of digital phenotyping of stress is that it might help us to understand how, for a given individual, stress is related to temporal features, how sleep impacts their stress levels, and how their stress relates to their smartphone app use. Identifying the latter relations might serve as a probe for qualitative investigation of how they respond to stress. For instance, if an individual spends less time on messenger apps when stressed, this could suggest that they avoid social contact, whereas seeking social support might be beneficial. If individuals are not aware of this pattern, personalized prediction models could provide novel clinical insights and could potentially help to improve therapy outcomes (eg, within personalized treatment modules [40]).

Finally, our exploratory findings provide important directions for confirmatory research. Contrary to the approach taken here, which is useful for discovering potential digital markers, a confirmatory approach would be to test a small number of (preregistered) hypotheses that make explicit what relation we expect between specific digital markers and momentary subjective stress. Confirmatory research is required to test the robustness of (1) the relation between stress and the potential digital markers identified here and (2) the interindividual variability in this relation.

### Limitations

This study should be viewed in light of the following limitations. First, our findings have constraints on generality, as they are based on a sample of students at a small university in the Netherlands and, therefore, are more likely to generalize to student than nonstudent populations. Furthermore, we measured these individuals during the COVID-19 pandemic, when the original (Wuhan) strain of the SARS-CoV-2 virus was dominant. As results suggest that the COVID-19 pandemic and resulting lockdown affected participant stress (generally increasing stress but decreasing stress in some individuals), potentially, these results might have been different had there not been a pandemic. We encourage future research to test if our results replicate in other populations (eg, working adults or individuals diagnosed with mental disorders) and during a period with a limited SARS-CoV-2 infection rate.

Second, it is conceivable that not all students self-reported stress accurately at every assessment. A significant proportion of variance might therefore represent noise that cannot be explained by any variable or model, irrespective of modeling decisions. This is especially an issue for idiographic models that rely on the participants’ final observations, which might be observations of lower quality because of study fatigue [41].

Third, we applied within-person mean centering to the self-reported stress. Although this corrects for differences in how people use a scale, it only allows nomothetic models to predict whether a person is currently experiencing more or less stress than usual and prevents models from predicting whether this person’s stress level is very low or high relative to other people’s stress level.

Fourth, we forced nomothetic models to learn one mapping function from features to outcome for all individuals in our sample. This is problematic because such models learn 1 set of parameters that might be accurate for some individuals but highly inaccurate for others (ie, *one-size-fits-all* fallacy) [42]. Truly idiographic models, which we also trained in this study, do not have this issue by default. However, this comes at the cost of strongly reduced sample size, which limits model complexity and may lead to overfitting. As collecting more self-report data per individual is not feasible for samples of this size, future studies could (1) focus on smaller samples with exceptionally motivated participants for a longer sampling period, (2) use wearables to measure psychophysiological signals of stress (eg, CortiWatch [43]), or (3) train machine learning models on a full data set without losing sight of interindividual differences in feature-outcome relations (eg, using transfer learning [44]).

### Conclusions

Our exploratory study has 3 main conclusions. First, temporal features, sleep proxies, and prolonged use of messenger and social network apps are consistently identified as the most important digital markers for predicting momentary subjective stress. Second, for most people (200/224, 89.3%), these markers are not sufficiently informative to recognize momentary subjective stress with appreciable improvements in accuracy over a baseline model but do produce predictions that correlate with subjective stress. Third, the utility and relation with stress of (some) digital markers varies from person to person. On the one hand, 1 digital marker may be relevant to momentary subjective stress in one individual but not in another. On the other hand, the increase of 1 digital marker might imply lower stress for one individual and higher stress for another. Our study thus provides evidence for phenotypic heterogeneity in the relation between how we feel and the digital traces we leave behind. These findings are relevant for the implementation of algorithms in mobile health apps to prevent, monitor, and treat stress-related mental health problems.

## Appendix

Multimedia Appendix 1 Description of the algorithm used to estimate sleep duration and onset from smartphone app use log data.

Multimedia Appendix 2 List of hyperparameters and the grid of hyperparameter values that were searched to optimize models in cross-validation.

Multimedia Appendix 3 Shapley additive explanations (SHAP) beeswarm plots for all model types and splits of the data.

Multimedia Appendix 4 Stacked bar plot of zero-order correlations between features and stress on a person-by-person basis.

## Abbreviations

CV: cross-validation
LASSO: least absolute shrinkage and selection operator
MAE: mean absolute error
RF: random forest
SHAP: Shapley additive explanations
SVR: support vector regression
