# Supplementary material for: Smartphone-Tracked Digital Markers of Momentary Subjective Stress in College Students: Idiographic Machine Learning Analysis
Source: JMIR Mhealth Uhealth. 2023 Mar 23;11:e37469. doi: 10.2196/37469 (PMC10132040; doi:10.2196/37469)
Supplement: Multimedia Appendix 1 [file mhealth_v11i1e37469_app1.docx]

*Description of sleep algorithm*

The sleep duration rule-based algorithm aims to estimate the total sleep duration by identifying the start and end point of sleep. These points are selected as the minimum, within a window, of a minute-by-minute rolling sum of phone use throughout the day. The following steps were followed for each day of data. First, we transformed the raw smartphone log data to a binary time series for each individual person. In this time-series, each value indicates if a person was on their smartphone (1) or not (0) at least once in a given minute. For instance, part of the time-series might look like this: 23:01 - 0, 23:02 - 0, 23:03 - 0, 23:04 - 1. This means that a person was not on their smartphone between 23:00 and 23:04 and on their smartphone between 23:04 and 23:05. Second, we then calculated the six hours of the day during which the individual shows the lowest smartphone activity using the time-series from step 1. Third, we then calculated a 2-hour centered moving sum (i.e., we took one minute and computed the total smartphone usage for the 60 minutes before and after) for the binary smartphone usage time-series. Fourth, we selected all 2-hour windows with less than 2 minutes of total smartphone use that fell within the individual’s 6 hours of least activity. Fifth, from these 2-hour windows, which all have a start and end time, we selected the minimal start time as putative sleep onset (i.e., the sleep onset proxy included in the analysis) and maximal end time as putative sleep offset. Finally, we then calculated putative sleep duration (i.e., the Sleep duration proxy included in the analysis) by subtracting sleep offset from sleep onset.
