# Supplementary material for: Smartphone-Tracked Digital Markers of Momentary Subjective Stress in College Students: Idiographic Machine Learning Analysis
Source: JMIR Mhealth Uhealth. 2023 Mar 23;11:e37469. doi: 10.2196/37469 (PMC10132040; doi:10.2196/37469)
Supplement: Multimedia Appendix 2 [file mhealth_v11i1e37469_app2.docx]

Table S1. Hyperparameters tuned during training. Hyperparameter names correspond with *sklearn* argument names.

| Model | Hyperparameter | Grid values | Minimized error metric |
| --- | --- | --- | --- |
| Support vector regression (RBF kernel) | Gamma | ["scale","auto"] | Epsilon-insensitive loss |
|  | C | [0.000001, 0.00001, 0.0001, 0.001, 0.01, 1, 10, 100, 1000] |  |
|  | Epsilon | [0.0001, 0.001, 0.01, 0.1, 0.3, 0.5, 0.7, 0.9, 1] |  |
| Random forest | Number of estimators | [10, 20, 50, 100, 200, 500, 1000, 2000] | MSE |
|  | Max depth | [1, 5, 10, 20, 40, 50, 100] |  |
|  | Max features | [2, 4, 6, 8, 10, 12, 14, 16, 18, 20] |  |
|  | Max leaf nodes | [2, 5, 10, 20, 40, 50, 100] |  |
|  | Min samples leaf | [0.001, 0.01, 0.1, 0.2, 0.3, 0.4, 0.5] |  |
|  | Min samples split | [0.001, 0.01, 0.1, 0.2, 0.3, 0.4, 0.5] |  |
|  | Min weight fraction leaf | [0.001, 0.01, 0.1, 0.2, 0.3, 0.4, 0.5] |  |
| LASSO | Alpha | 0.000001, 0.00001, 0.0001, 0.001, 0.01, 0.1, 1, 10, 100, 1000, 10000, 100000 | MSE with L1 penalty |
