# Supplementary material for: Smartphone-Tracked Digital Markers of Momentary Subjective Stress in College Students: Idiographic Machine Learning Analysis
Source: JMIR Mhealth Uhealth. 2023 Mar 23;11:e37469. doi: 10.2196/37469 (PMC10132040; doi:10.2196/37469)
Supplement: Multimedia Appendix 3 [file mhealth_v11i1e37469_app3.docx]

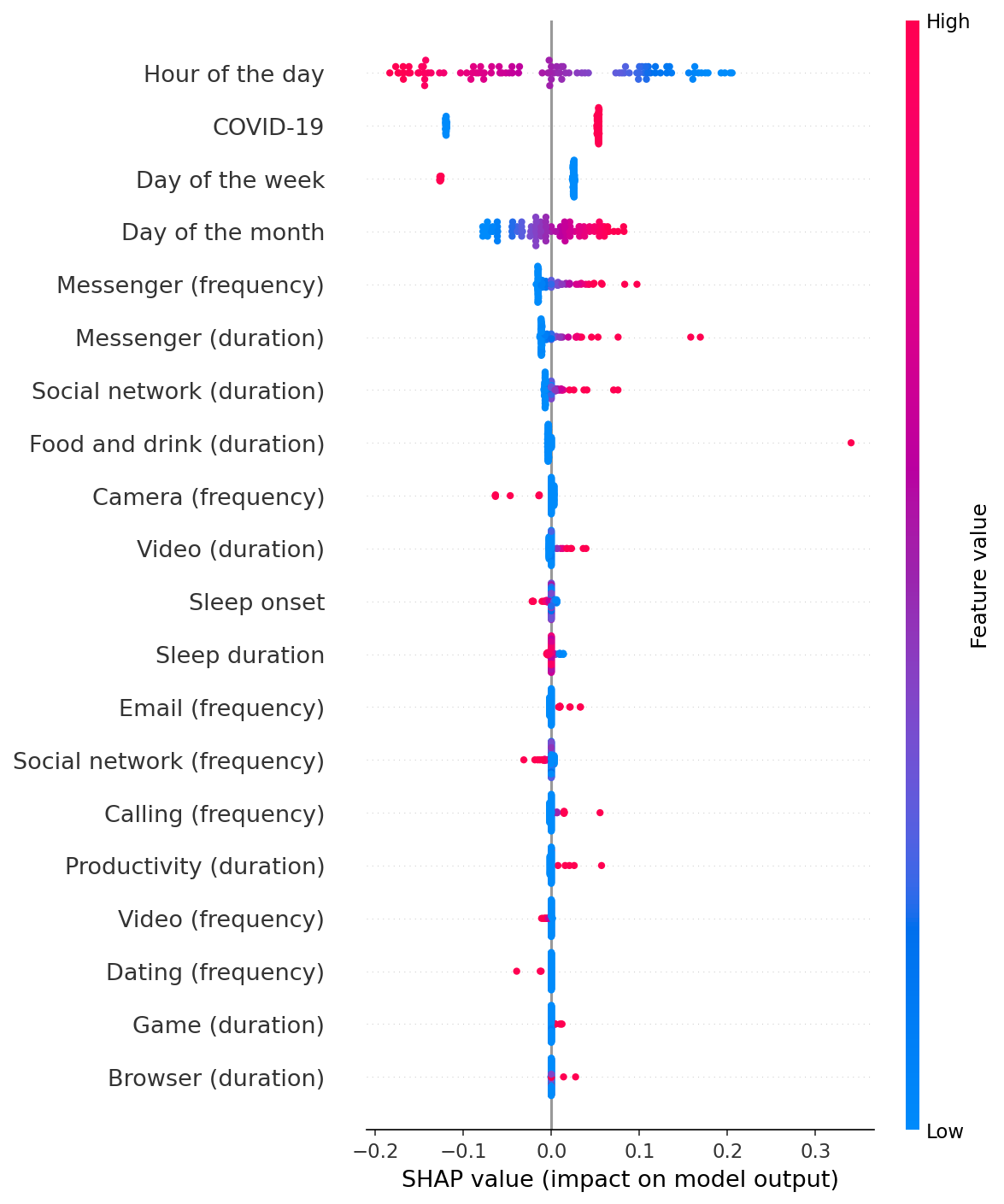


Figure S1. Beeswarm plot for the nomothetic LASSO regression in data split 1.


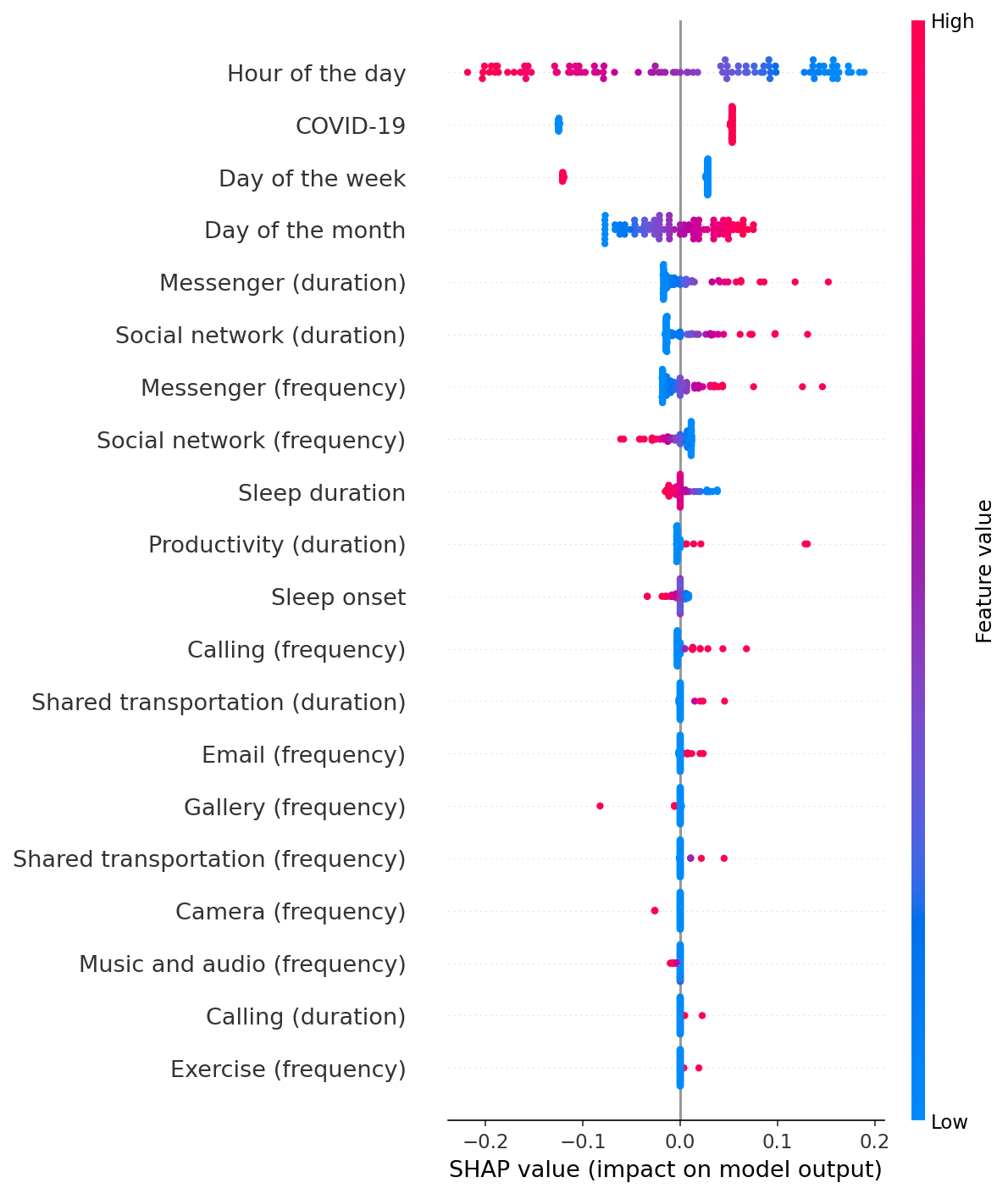


Figure S2. Beeswarm plot for the nomothetic LASSO regression in data split 2.


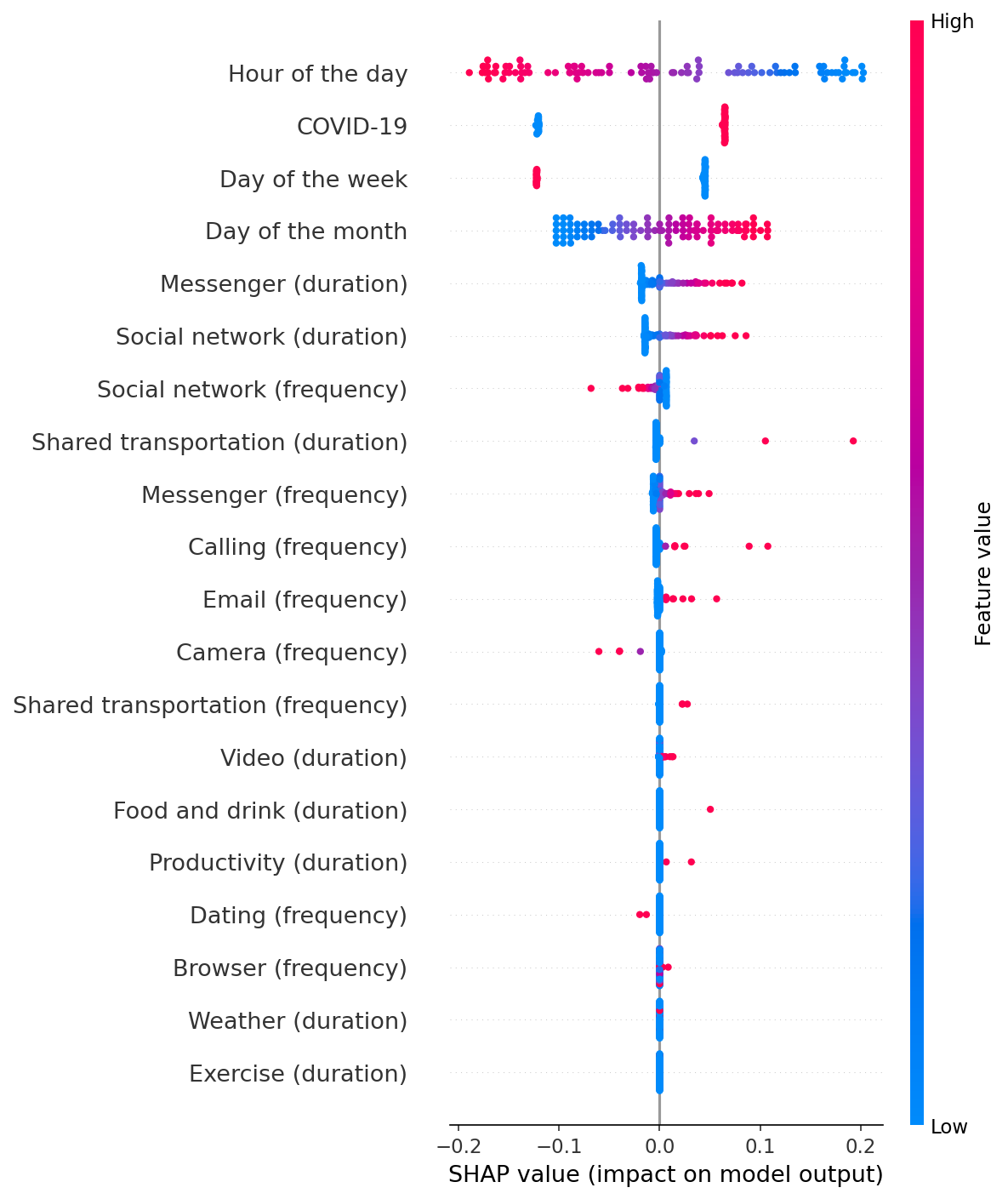


Figure S3. Beeswarm plot for the nomothetic LASSO regression in data split 3.


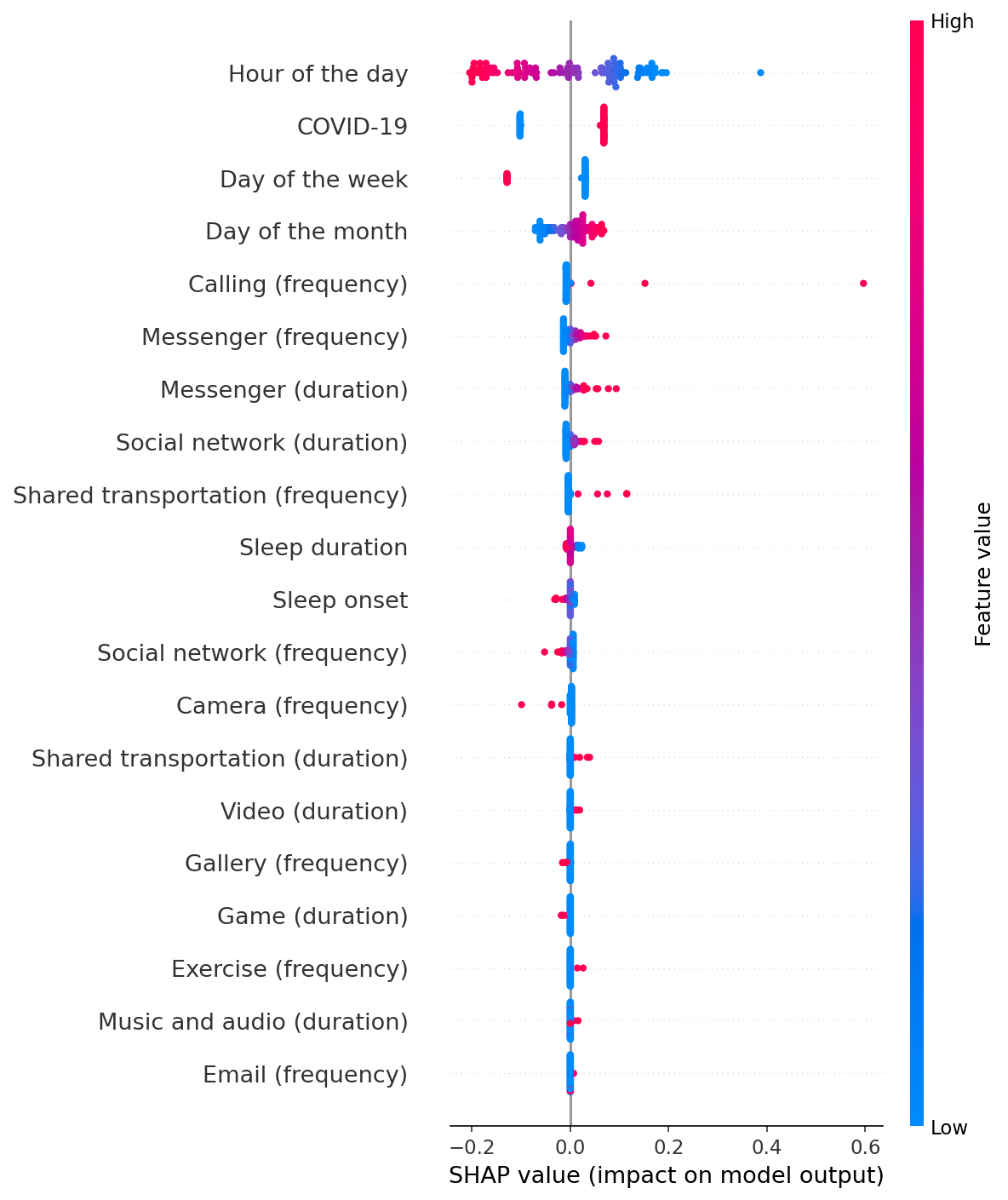


Figure S4. Beeswarm plot for the nomothetic LASSO regression in data split 4.


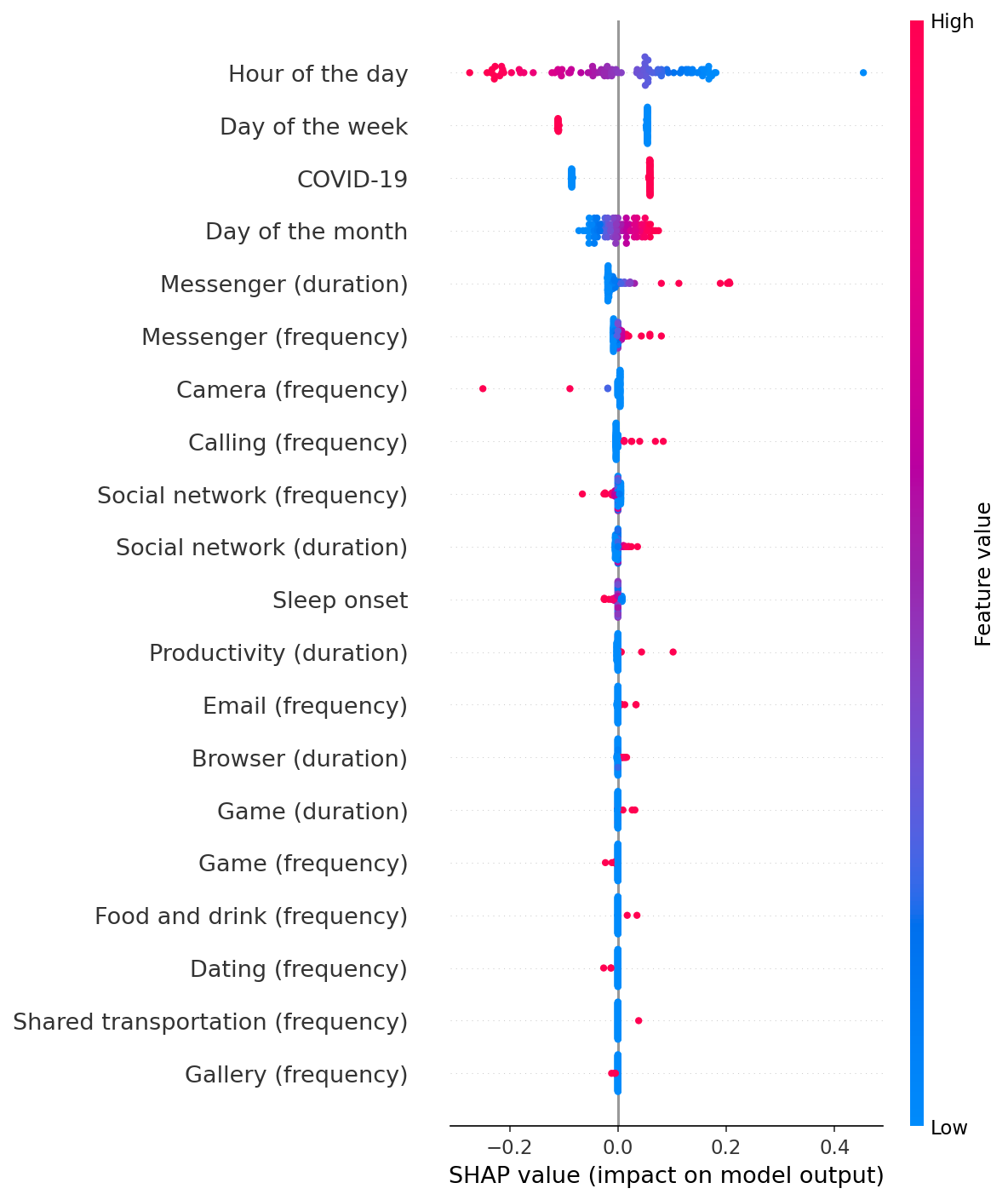


Figure S5. Beeswarm plot for the nomothetic LASSO regression in data split 5.


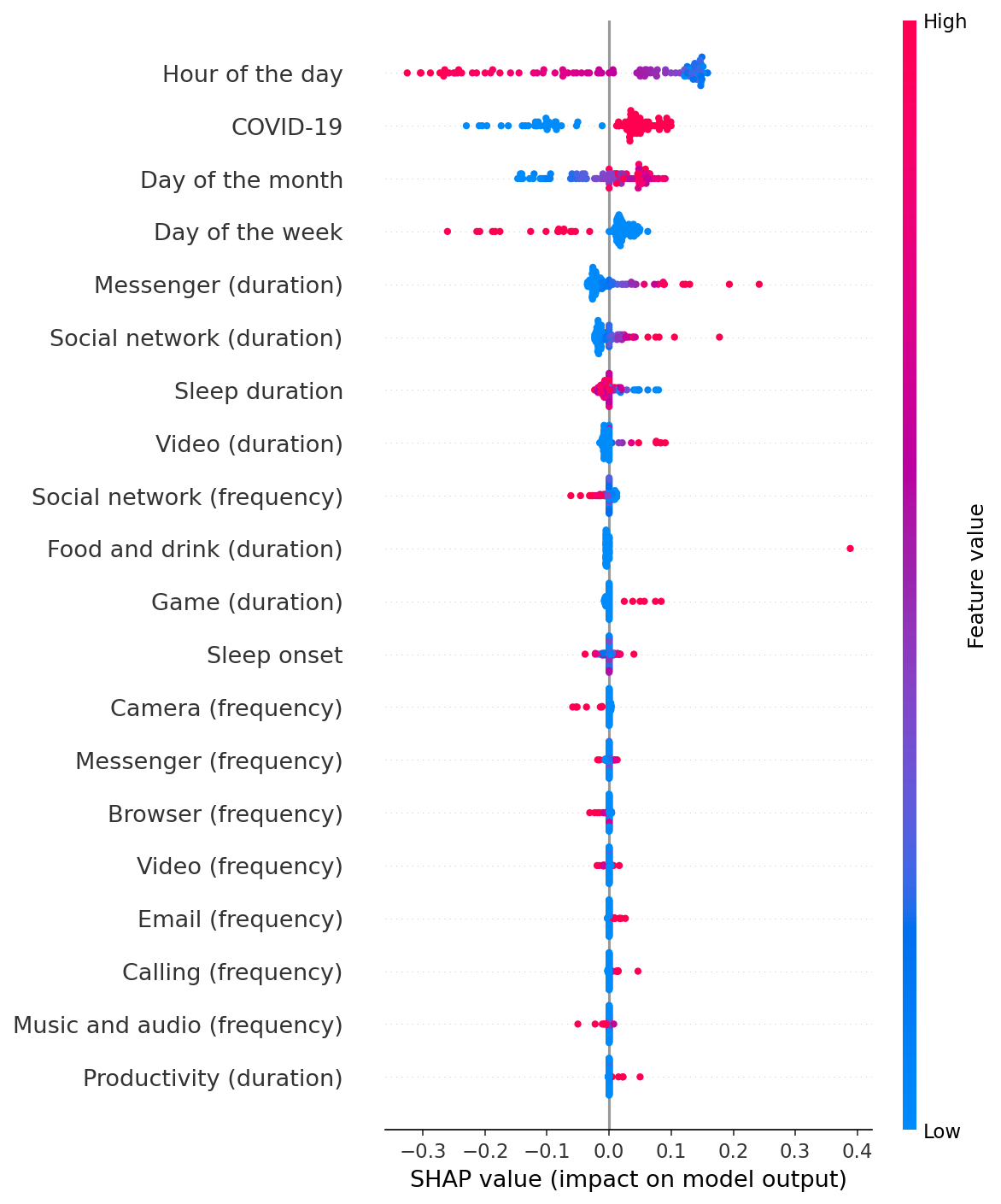


Figure S6. Beeswarm plot for the nomothetic support vector regression in data split 1.


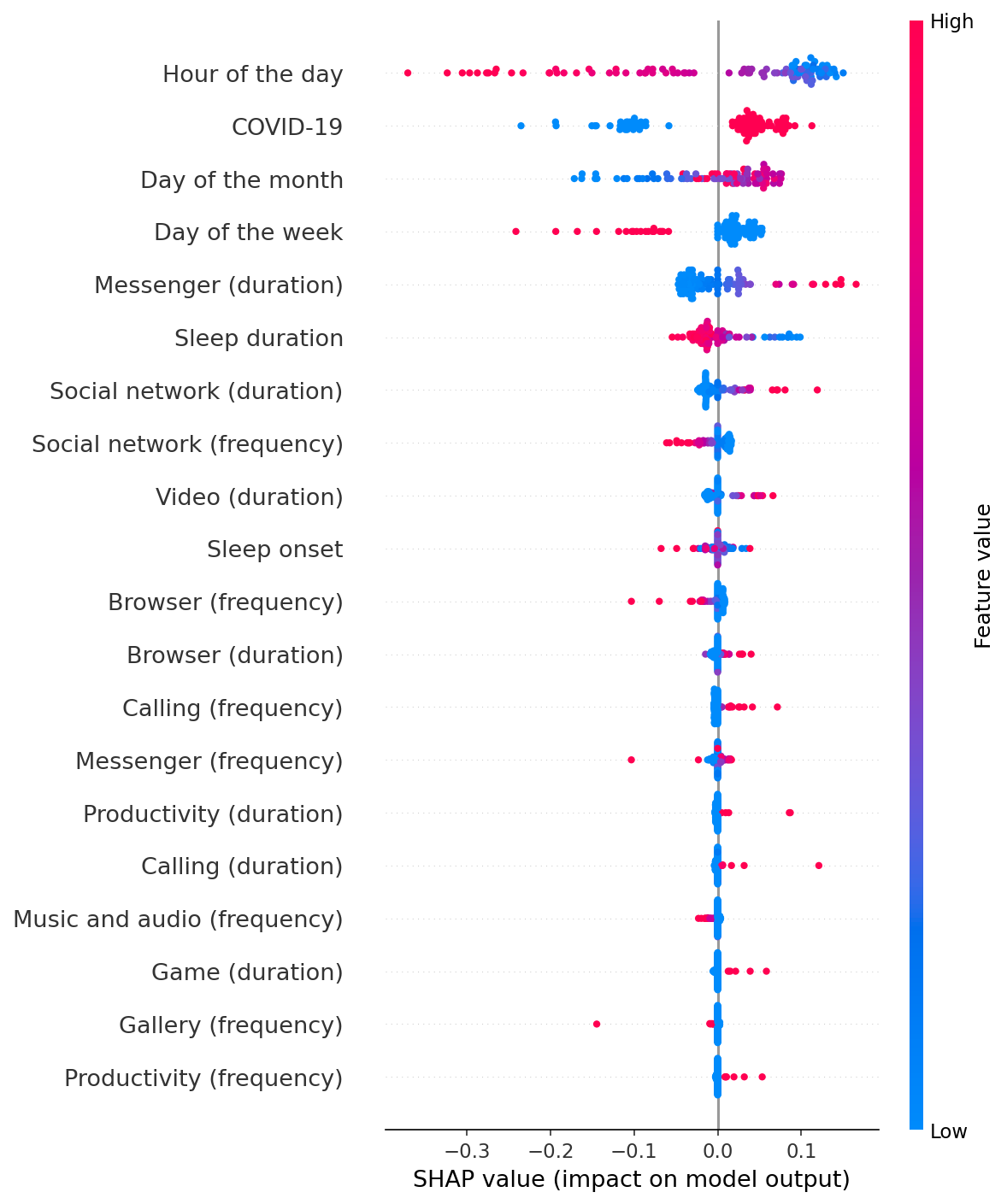


Figure S7. Beeswarm plot for the nomothetic support vector regression in data split 2.


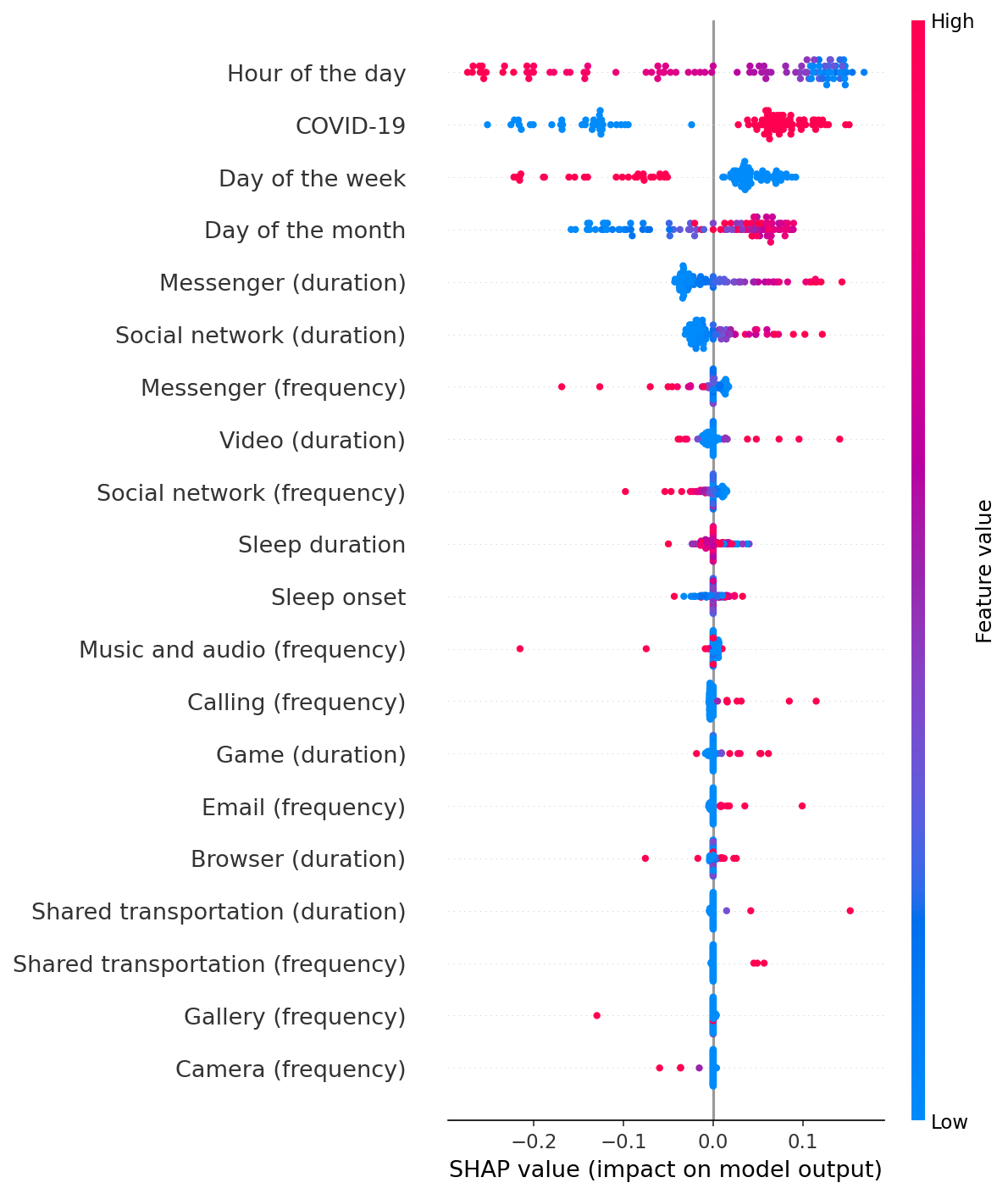


Figure S8. Beeswarm plot for the nomothetic support vector regression in data split 3.


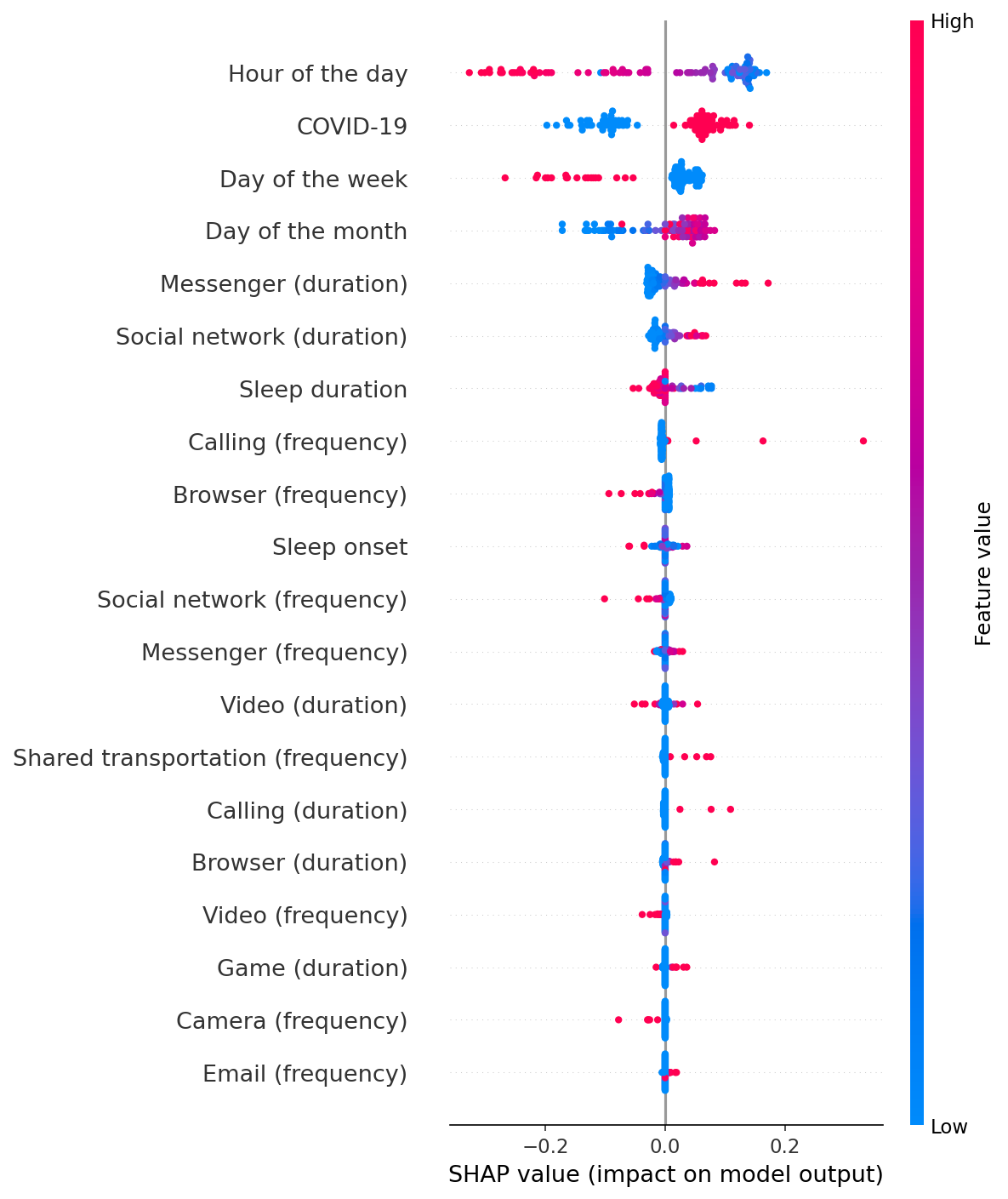


Figure S9. Beeswarm plot for the nomothetic support vector regression in data split 4.


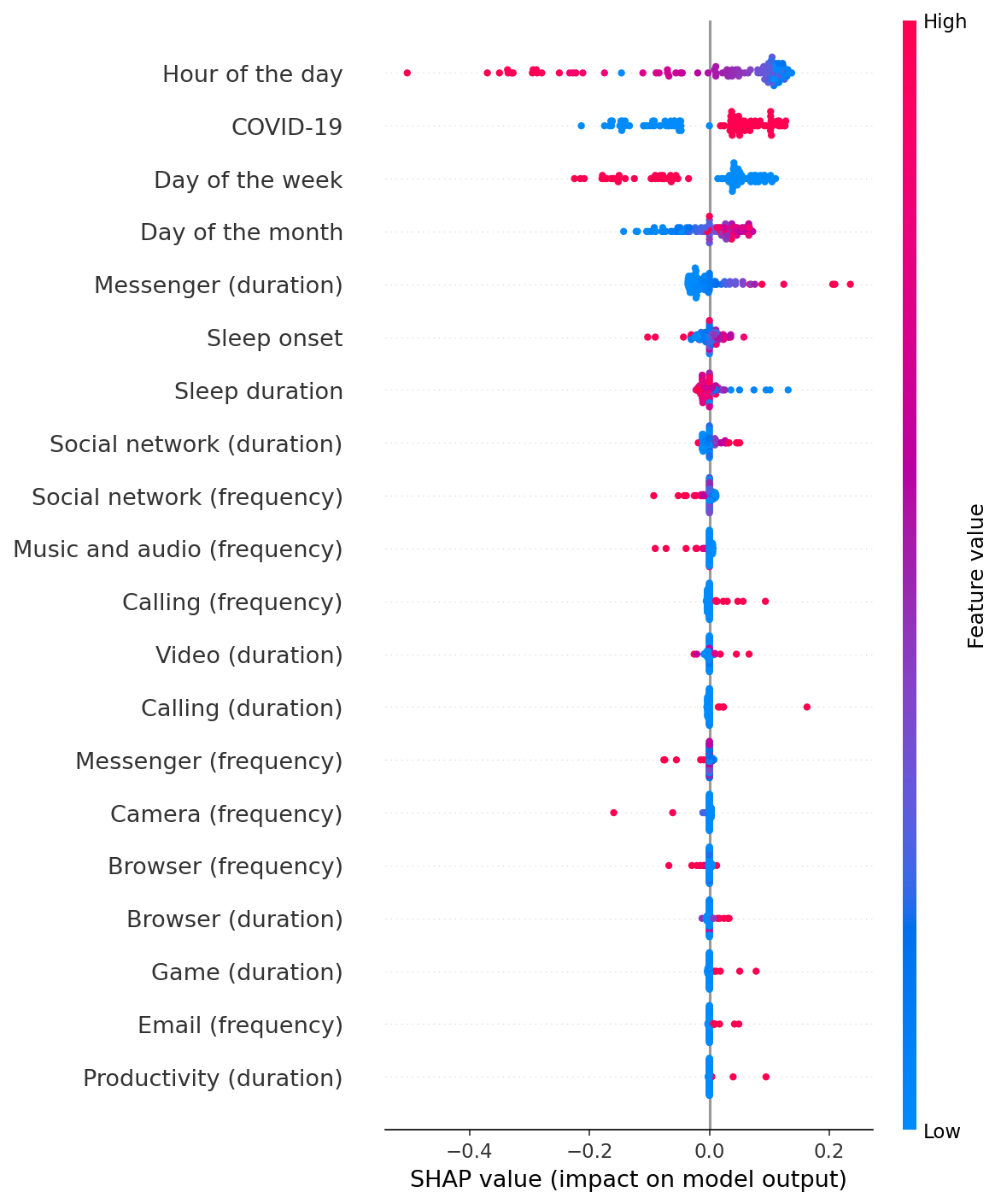


Figure S10. Beeswarm plot for the nomothetic support vector regression in data split 5.


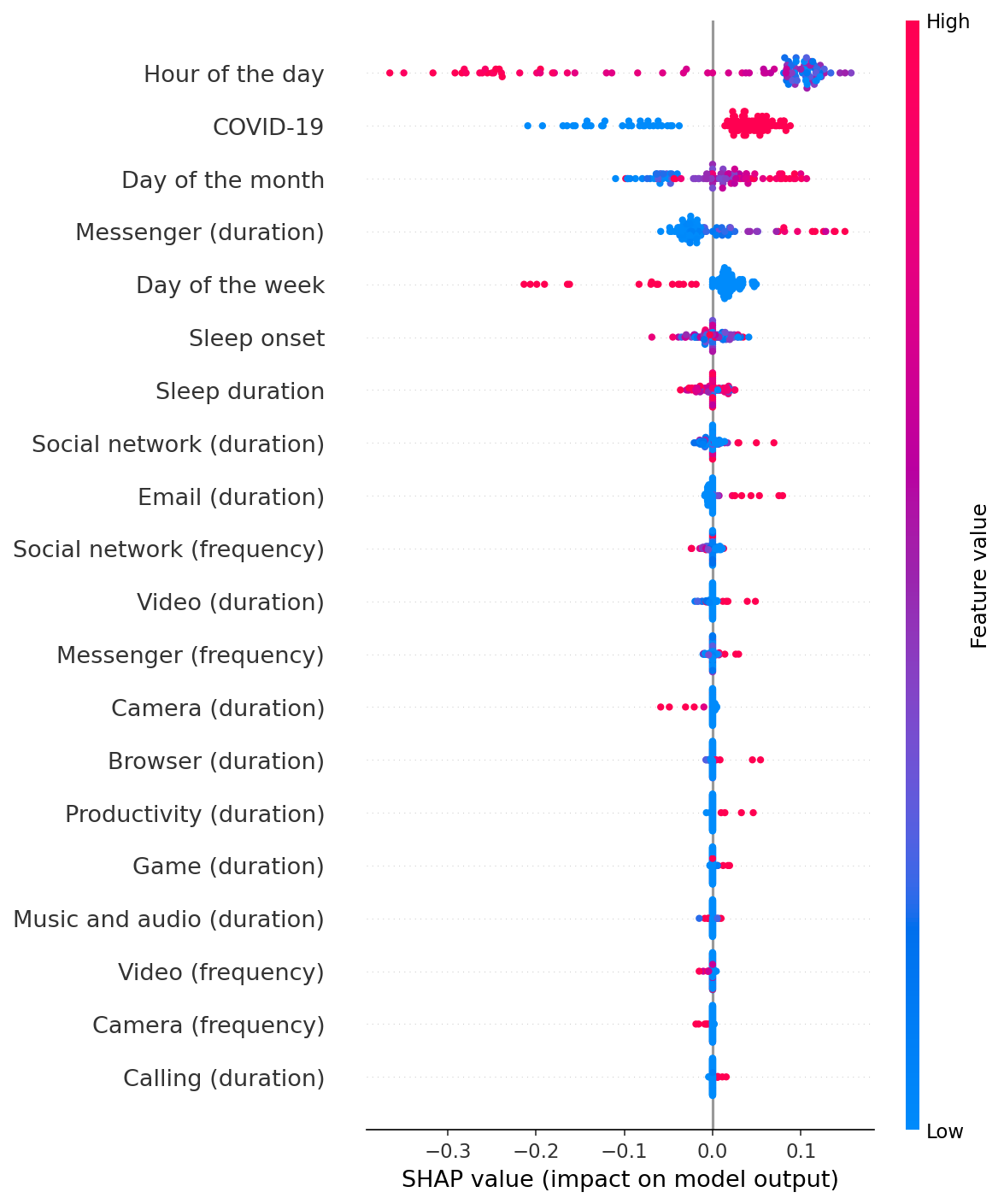


Figure S11. Beeswarm plot for the nomothetic random forest regression in data split 1.


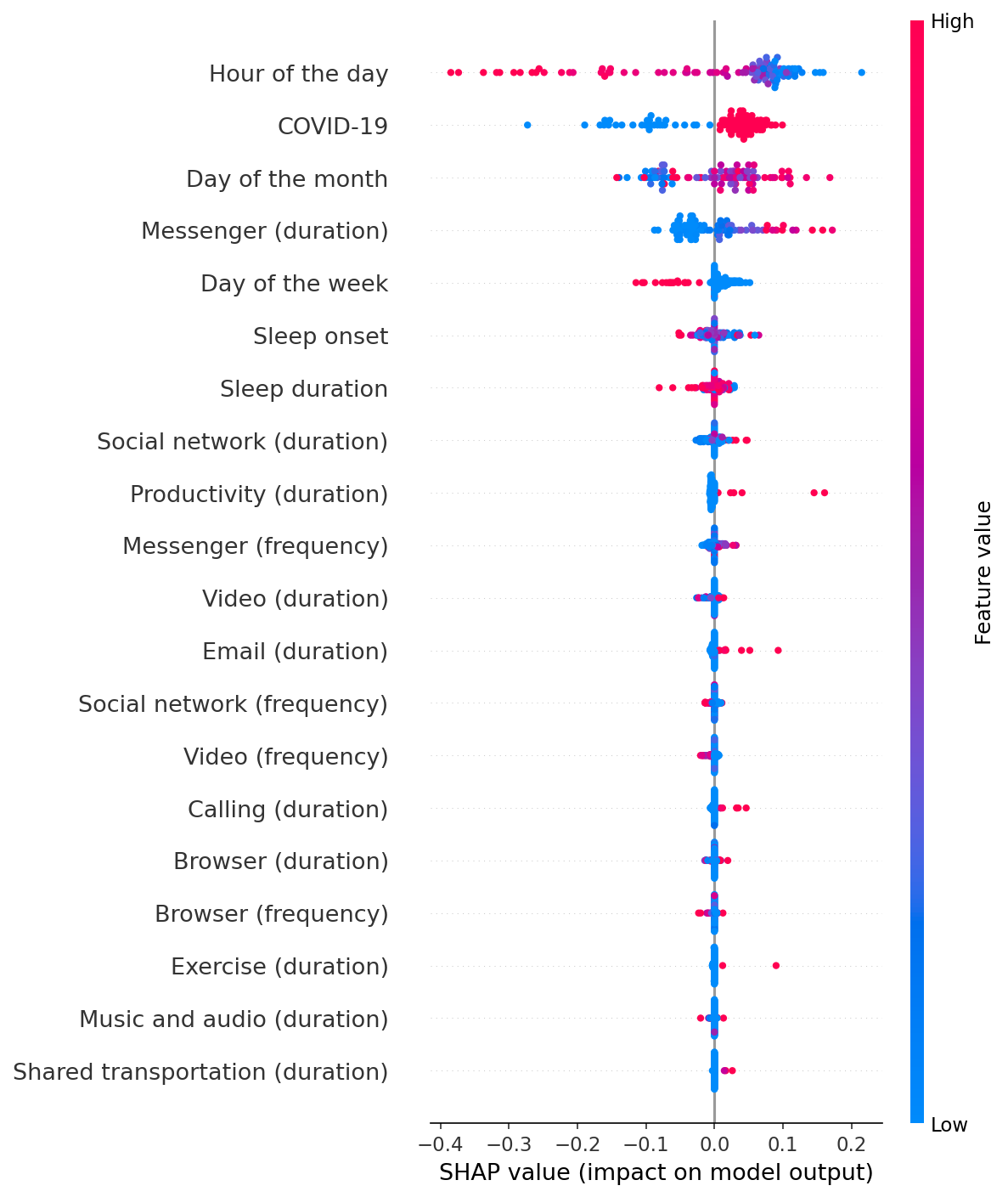


Figure S12. Beeswarm plot for the nomothetic random forest regression in data split 2.


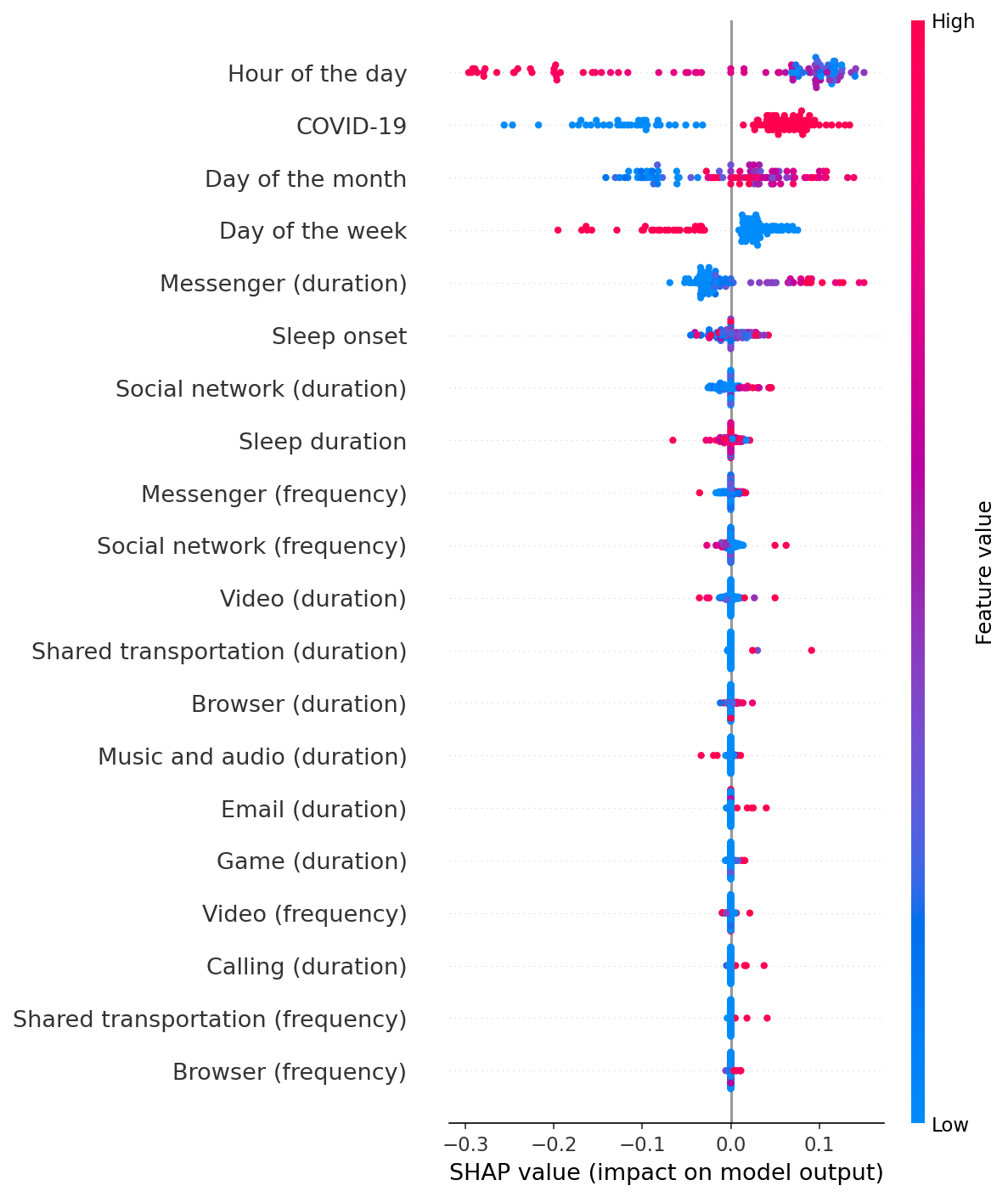


Figure S13. Beeswarm plot for the nomothetic random forest regression in data split 3.


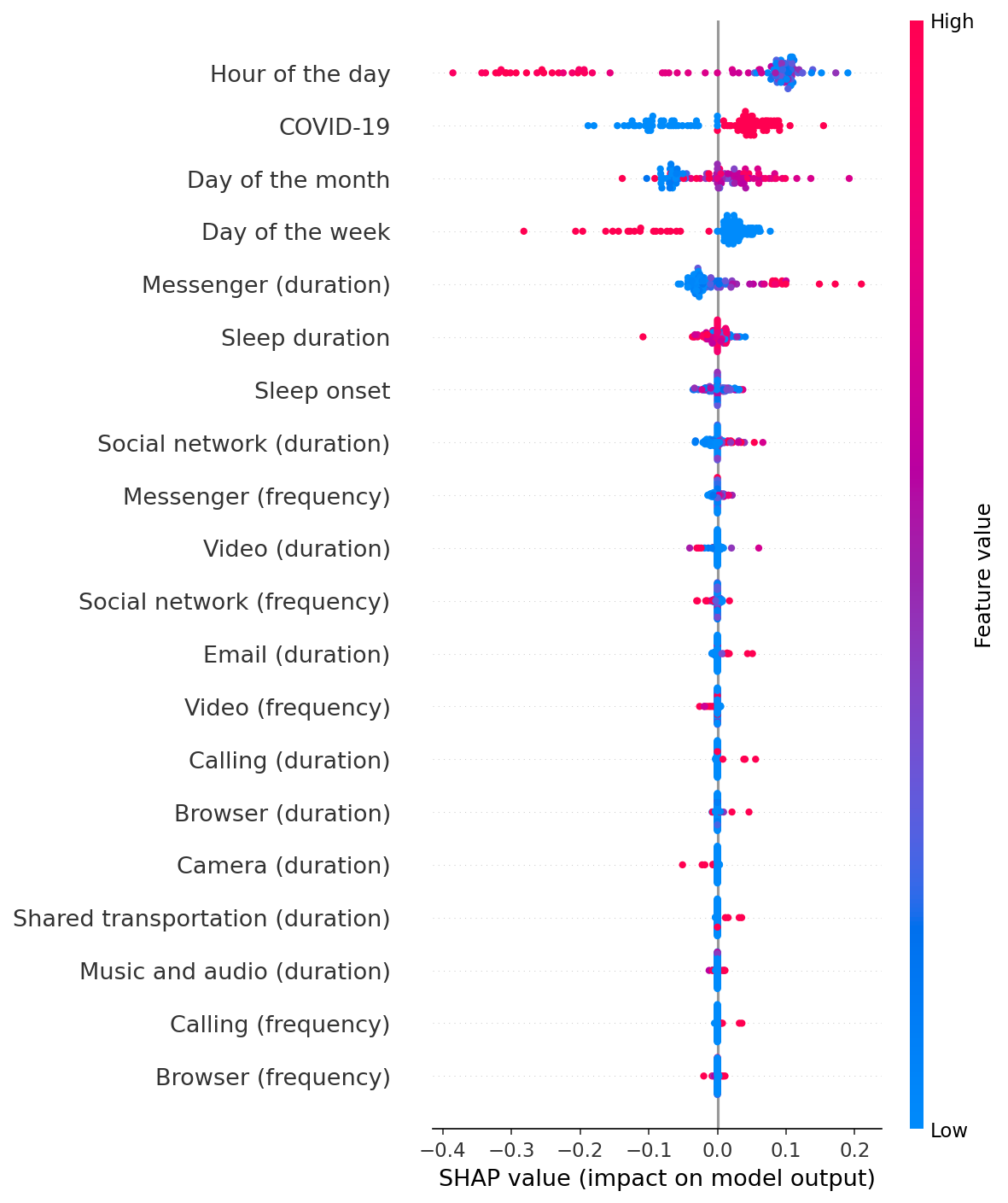


Figure S14. Beeswarm plot for the nomothetic random forest regression in data split 4.


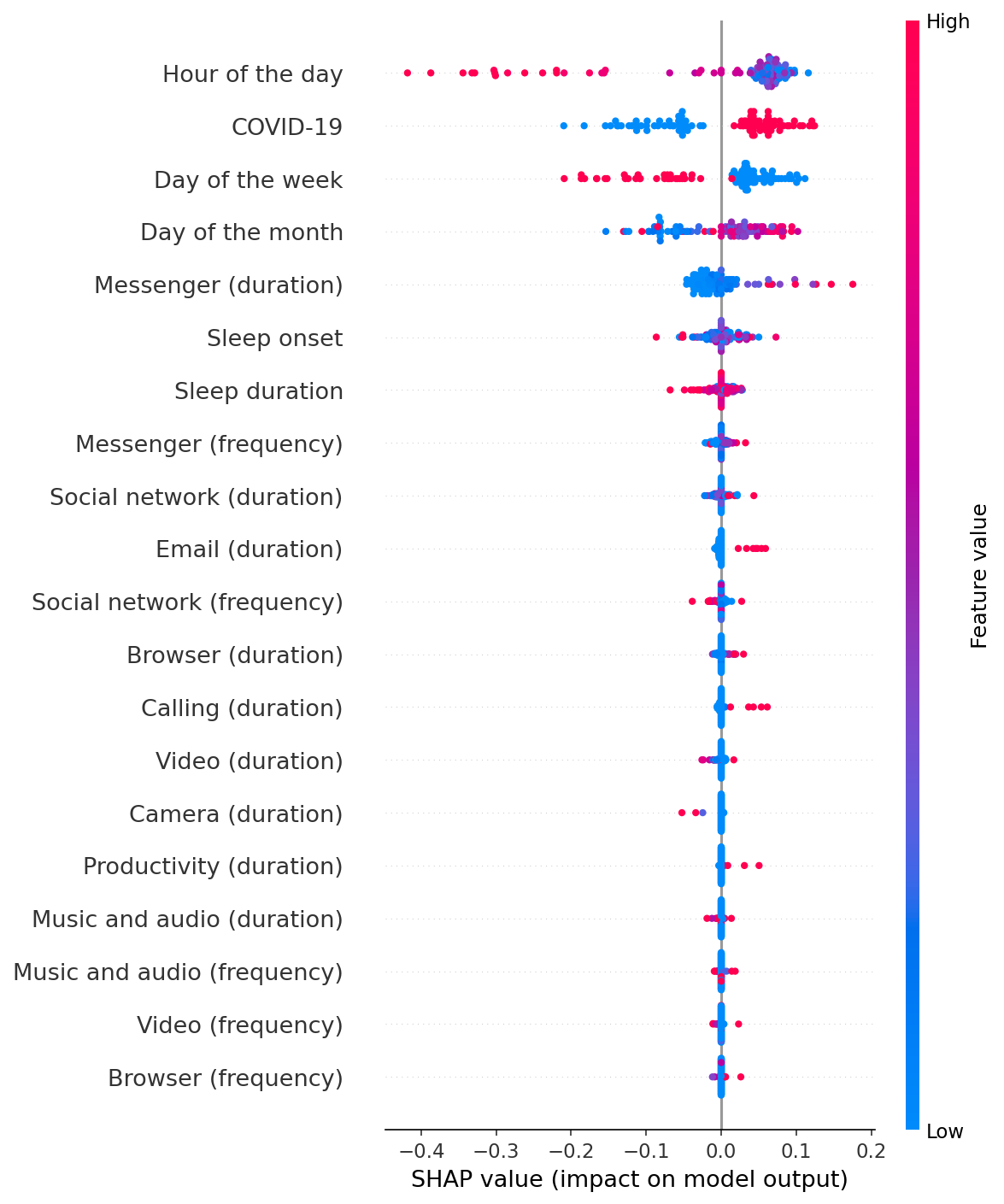


Figure S15. Beeswarm plot for the nomothetic random forest regression in data split 5.
