# Supplementary material for: Smartphone-Tracked Digital Markers of Momentary Subjective Stress in College Students: Idiographic Machine Learning Analysis
Source: JMIR Mhealth Uhealth. 2023 Mar 23;11:e37469. doi: 10.2196/37469 (PMC10132040; doi:10.2196/37469)
Supplement: Multimedia Appendix 4 [file mhealth_v11i1e37469_app4.docx]

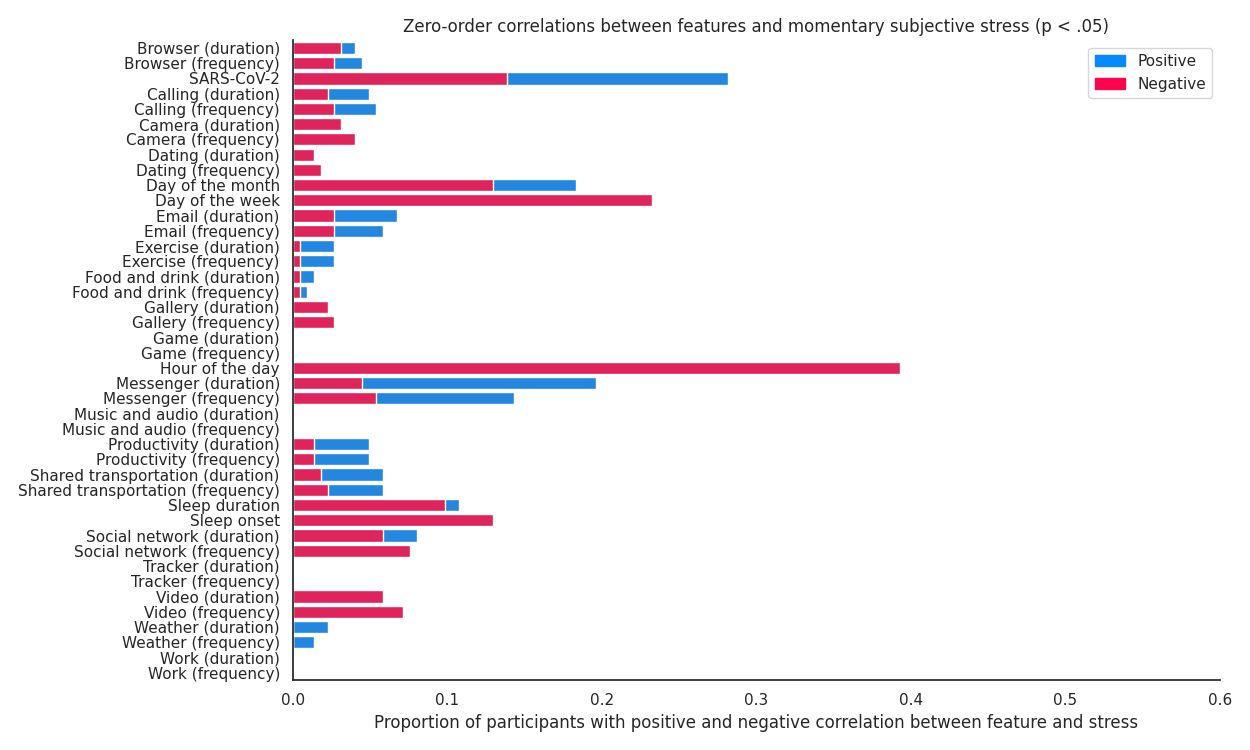


Figure S1. Stacked barplot visualizing the proportion of statistically significant positive and negative person-specific correlations between individual features and momentary subjective stress.
